# Reducing SARS-CoV-2 pathological protein activity with small molecules

**DOI:** 10.1016/j.jpha.2021.03.012

**Published:** 2021-04-05

**Authors:** Donata Pluskota-Karwatka, Marcin Hoffmann, Jan Barciszewski

**Affiliations:** a Faculty of Chemistry, Adam Mickiewicz University in Poznań, 61-614, Poznań, Poland; b NanoBiomedical Center of the Adam Mickiewicz University, 61-614, Poznań, Poland; c Institute of Bioorganic Chemistry of the Polish Academy of Sciences 61-704, Poznań, Poland

**Keywords:** SARS-CoV-2, COVID-19, Deamination, APOBEC, Therapeutic nucleotides, Natural products, Curcumin

## Abstract

Coronaviruses are dangerous human and animal pathogens. The newly identified coronavirus SARS-CoV-2 is the causative agent of COVID-19 outbreak, which is a real threat to human health and life. The world has been struggling with this epidemic for about a year, yet there are still no targeted drugs and effective treatments are very limited. Due to the long process of developing new drugs, reposition of existing ones is one of the best ways to deal with an epidemic of emergency infectious diseases. Among the existing drugs, there are candidates potentially able to inhibit the SARS-CoV-2 replication, and thus inhibit the infection of the virus. Some therapeutics target several proteins, and many diseases share molecular paths. In such cases, the use of existing pharmaceuticals for more than one purpose can reduce the time needed to design new drugs. The aim of this review was to analyze the key targets of viral infection and potential drugs acting on them, as well as to discuss various strategies and therapeutic approaches, including the possible use of natural products. We highlighted the approach based on increasing the involvement of human deaminases, particularly APOBEC deaminases in editing of SARS-CoV-2 RNA. This can reduce the cytosine content in the viral genome, leading to the loss of its integrity. We also indicated the nucleic acid technologies as potential approaches for COVID-19 treatment. Among numerous promising natural products, we pointed out curcumin and cannabidiol as good candidates for being anti-SARS-CoV-2 agents.

## Highlights

• Human APOBEC deaminases may reduce the cytosine content in the SARS-CoV-2 genome.
• Human APOBEC deaminases may cause the loss of SARS-CoV-2 genome integrity.
• Strategies based on nucleic acid technologies can be useful for COVID-19 treatment.
• Natural products can be a base for specific drugs against SARS-CoV-2 development.
• Curcumin and cannabidiol are good candidates for being anti-SARS-CoV-2 agents.

## Graphical abstract

**Figure:**
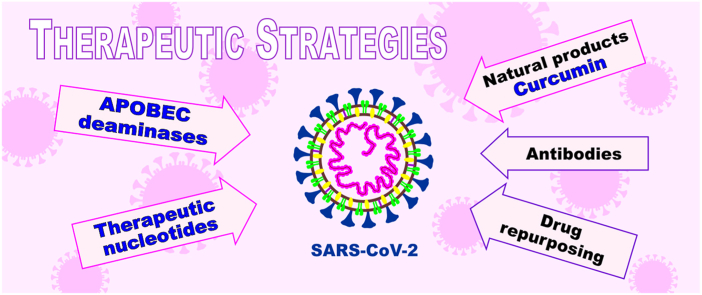

## 1 Introduction

The first coronavirus, the infectious bronchitis virus (IBV), isolated from chicken embryos, was described in the 1930s [1], and the first coronaviruses infecting humans, designated HCoV-229E and HCoV-OC43, were reported in the 1960s [2,3]. Since the discovery of the first family member, coronaviruses have attracted more and more attention from around the world. This is due to emergence of the dangerous human diseases caused by three viruses of the family; severe acute respiratory syndrome coronavirus 1 (SARS-CoV-1), Middle East respiratory syndrome coronavirus (MERS-CoV), and lately identified severe acute respiratory syndrome coronavirus 2 (SARS-CoV-2) [4]. In humans, coronaviruses are responsible for infection of upper and lower respiratory tracts [5]. In most individuals symptoms evoked by HCoV-229E, HCoV-OC43, HCoV-HKU1 and HCoV-NL63 are mild [6,7], and hence until 2002 coronaviruses were considered relatively simple nonfatal pathogens. The approach to coronaviruses radically changed at the end of 2002 when SARS-CoV-1 and severe acute respiratory syndrome (SARS) caused by it appeared [8]. The world was completely surprised by emergence of coronavirus causing not typical severe pneumonia, and shocked by the outbreak of such a disease in 21st century [9,10]. With the advent of another coronavirus (MERS-CoV) in 2012, also responsible for severe pneumonia in humans [11], it became clear that such pathogens could pose a very serious threat to health [10]. MERS, besides sharing with SARS, such a clinical feature as severe atypical pneumonia, is also associated with acute renal failure and gastrointestinal problems [12]. SARS-CoV-1 and MERS-CoV caused epidemics which had considerable associated mortality of about 10% and 35%, respectively (MERS-CoV is still active) [13]. In late fall of 2019, a novel acute respiratory disease, coronavirus disease 2019 (COVID-19) appeared, the origin of which has not yet been established. It is more transmissible than SARS and MERS, characterized by asymptomatic carriers, long latency period, high infectivity, and relatively high mortality of about 5% [13,14]. The disease is caused by SARS-CoV-2, which like SARS-CoV-1 and MERS-CoV, primarily targets the lower respiratory system. However, apart from causing pneumonia, infection with SARS-CoV-2 may also result in the failure of various organ functions [15]. The immune response to SARS-CoV-2 infection can be extreme, causing a sharp rise in inflammatory cytokines and chemokines that may contribute to the onset of a life-threatening complication known as cytokine storm syndrome [16]. Mortality associated with COVID-19 is unclear due to diverse procedures used in different countries to track the cases; however, spread of the disease throughout the world is unprecedentedly rapid. Therefore, there is an urgent need for development of the appropriate treatment. Meanwhile, although it is the third life threatening disease caused by a virus from this family, no targeted drugs are currently available for the treatment and prevention of COVID-19. Yet development of targeted drugs is time consuming and so far, life supportive care and symptomatic treatment remain the only alternative. Repurposing of existing therapeutics and strategies seems to be the best option for treating the clinical symptoms of the infection such as cough, fever and difficulty in breathing, to combat the disease. Apart from therapies based on commercially available pharmaceuticals, approaches using natural metabolites and therapeutic nucleotides as well as plasma and monoclonal antibodies seem to be interesting strategies. In this work, we review these strategies and analyze drugs involved in them, along with their viral and host cellular targets.

## 2 *Coronaviridae* family

*Coronaviridae* family includes enveloped, positive-sense, single-stranded RNA viruses belonging to three vertebrate classes: mammals (corona- and toroviruses), birds (coronaviruses) and fish (bafiniviruses). In *Coronavirinae* virions are spherical. They can also be rod-shaped like in bafinivirus or constitute a mixture of both, with rod-shaped particles bent into crescents (torovirus). The particles are decorated with surface projections (“peplomers” or “spikes”). In electron micrographs, the spherical shape generates an image resembling the solar corona. Therefore the name “coronaviruses” was coined. *Coronaviridae* family belongs to *Cornidovirineae* suborder belonging to the order named Nidovirales [17,18]. Phylogenetically Nidovirales are subdivided into alpha, beta, delta and gammacoronaviruses. Alpha and betacoronaviruses are the human pathogens [19]. So far, seven coronaviruses have been identified to infect humans. Apart from the betacoronaviruses responsible for severe diseases (SARS-CoV-1, MERS-CoV, and SARS-CoV-2), there are four others (HCoV-229E, HCoV-OC43, HCoV-NL63 and HCoV-HKU1), which cause common cold [13,20].

**Fig. 1:**
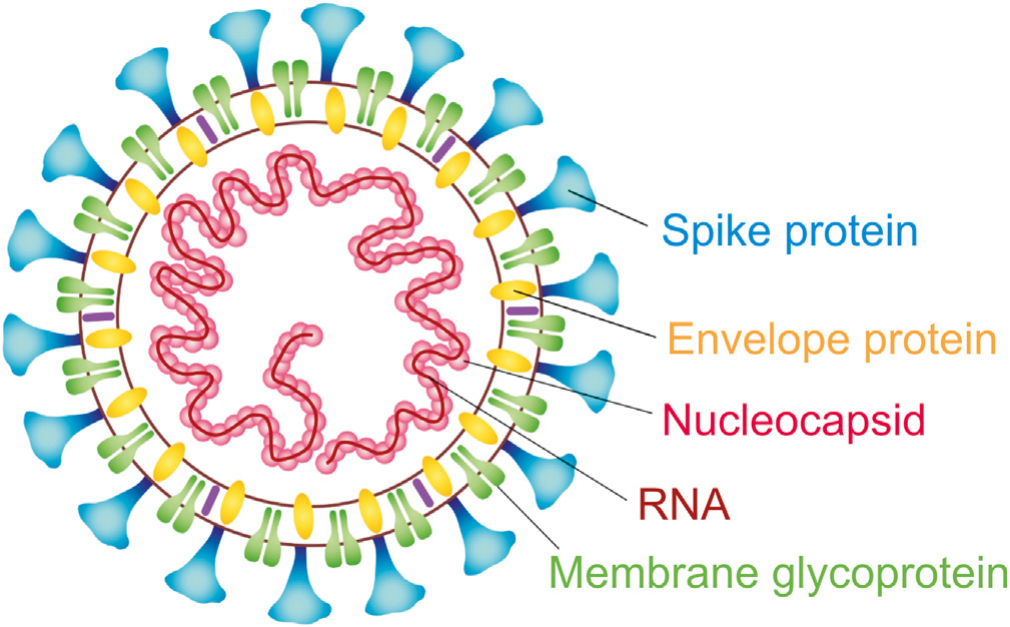
Structure of SARS-CoV-2. The main structural proteins and genetic material (RNA) are indicated.

Among RNA viruses, coronaviruses are believed to possess the largest genomes (27–32 kb) [18], containing several open reading frames (ORF) apart from two polyproteins, code structural and accessory proteins. Coronaviruses include members which can cross the barrier between species, causing the infection of a new species. Coronavirus particles consist of four structural proteins: spike (S), envelop (E), nucleocapsid (N) and membrane (M) (Fig. 1). Three of them: S, M and E, constitute membrane-associated proteins. Some betacoronaviruses include an additional membrane protein that exhibit hemagglutinin and esterase activities [21]. S protein is the glycosylated protein involved in viral binding to host cells and fusion. The most abundant protein is M. Due to the presence of three hydrophobic domains, the protein is tightly associated with the virus envelope. M protein plays a major role in promoting membrane curvature and interacts with the N and E proteins. The E protein is present in small amounts in the virion, while its bigger amounts can be found in infected cells. E protein was shown to assemble in membranes to form ion channels. The N protein is the only one present in the ribonucleoprotein particle. It binds genomic RNA and packages it into a long flexible nucleocapsid. N protein is also involved in other processes such as modulation of cell-cycle and inhibition of host cell translation. The hemagglutinin-esterase (HE) is glycoprotein projected outwards from the virion. HE binds sialic acid units on glycoproteins and glycolipids, and removes acetyl groups from O-acetylated sialic acid, and thus may be a receptor-destroying enzyme [21].

Coronaviruses also contain other components such as nonstructural proteins and proteins from the host cell. They are diverse and complex pathogens, which, despite this diversity, exhibit some common features.

## 3 Structure and life cycle of SARS-CoV-2

As a member of the *Coronaviridae* family, SARS-CoV-2 shares many of its defining characteristics: it is the positive-sense single-stranded RNA virus, spherical in form and characterized by club-shaped glycoprotein projections on its surface (Fig. 1).

Apart from the typical four main structural proteins, S, E, M and N, for coronaviruses belonging to this family, SARS-CoV-2 contains several accessory proteins [22].

**Fig. 2:**
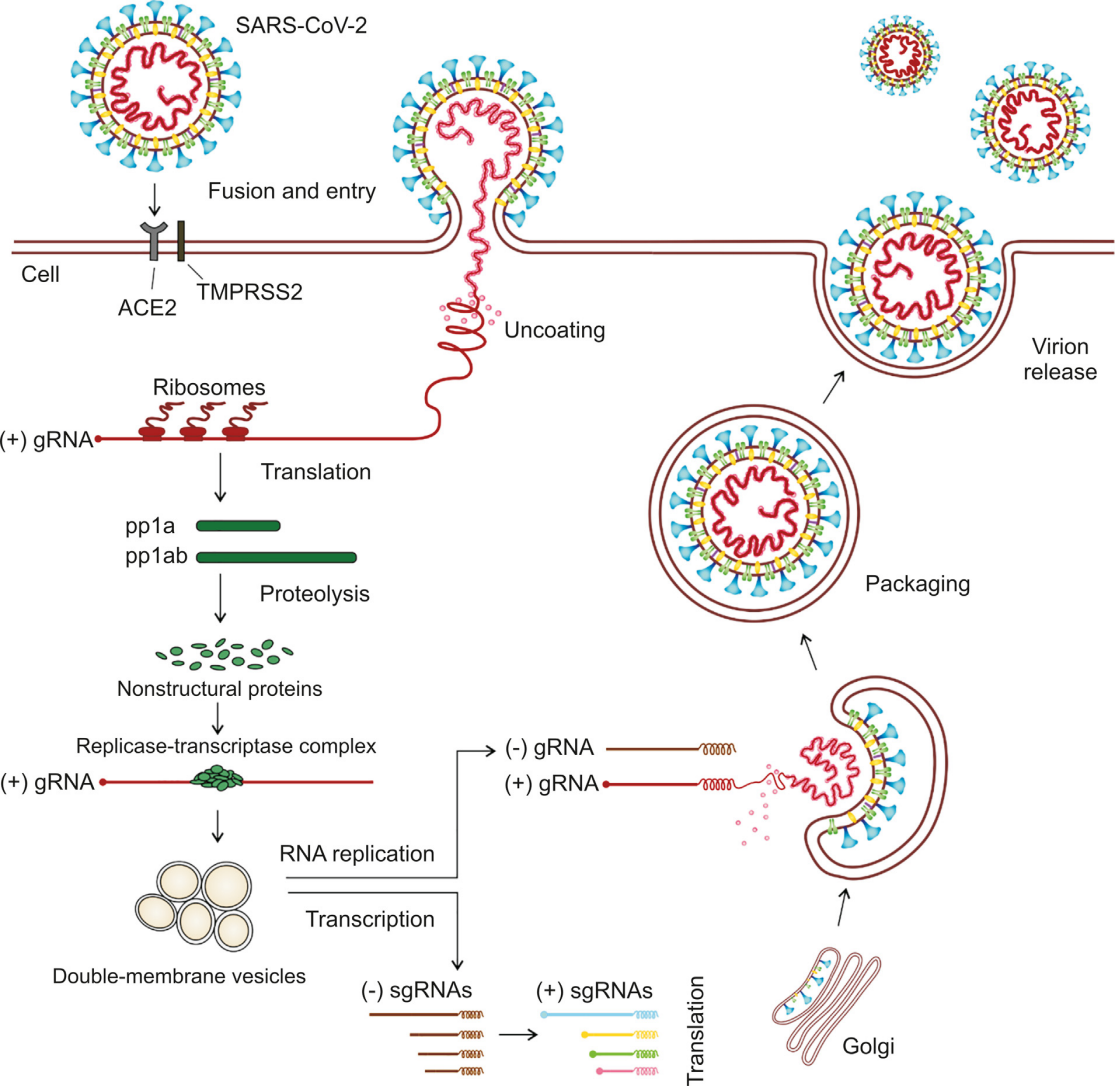
Simplified presentation of the SARS-CoV-2 life cycle. SARS-CoV-2 begins its life cycle through binding to the host cell receptor ACE2 (angiotensin converting enzyme 2) followed by the virus membrane fusion with the host cell membrane, activation of the attached S protein by transmembrane serine protease 2 and entry into the cell. Then SARS-CoV-2 releases its RNA which is translated into the two polyproteins pp1a and pp1ab. The polyproteins undergo proteolysis yielding numerous viral nonstructural proteins. After formation of replicase-transcriptase complex, a series of subgenomic RNAs are produced by transcription and finally translated into the viral structural proteins. In the ER (endoplasmic reticulum) and Golgi, virions are formed by assembling the viral structural proteins with genome. The virions are then transported and released from the cell.

In the SARS-CoV-2, life cycle following stages can be distinguished: fusion and entry, translation, proteolysis, translation and RNA replication, packaging and virion release (Fig. 2).

Initiating of the entry into the host cell, the viral Spike protein with binding domain located at 331 to 524 residues, interacts with human angiotensin converting enzyme 2 (ACE2), and attaches to it with high affinity using it as an entry receptor [23,24]. The viral membrane then fuses with the host cell plasma membrane. The process is followed by activation of the attached S protein by transmembrane serine protease 2 [23]. The activation results in conformational changes that allow the virus to enter the cell through endocytosis. RNA of the viral genome (gRNA) is then inserted into the cytoplasm. This process is accomplished by proteolytic cleavage of S protein [25].

With respect to the entry receptor, the recent studies showed that ACE2 is expressed to a limited extent in the upper respiratory tract, while in the lungs there is no or only very low expression of this enzyme [26]. Nevertheless, it is the cells of the respiratory system that are the target of SARS-CoV-2 attack, and the ease of its spreading does not correlate with low ACE2 expression in these cells, assuming that this enzyme is the main receptor for entry of the virus into the host cell. Lately, it was hypothesized that there are factors that facilitate the interactions of the virus with cells with low ACE2 expression, and it was shown that neuropilin-1 may be such a factor [27]. This discovery provides new information on the penetration of SARS-CoV-2 into host cells and on viral infectivity, at the same time indicating a potential new therapeutic target and strategy.

The key stage of the SARS-CoV-2 life cycle contains replication of its genome, occurring within the infected cells. This is a complex process of carrying out RNA polymerisation, proofreading and capping, in which various viruses and host proteins are involved. The positive polarisation of SARS-CoV-2 RNA makes it similar to mRNA. Due to this similarity, viral RNA can be read directly by ribosomes of the host cell. Two-thirds of the viral RNA located in the cytoplasm undergoes translation by the host cell protein translation system, into two large polyproteins pp1a and pp1ab. The remainder part is subjected to transcription into a set of subgenomic RNAs [28]. The two polyproteins are then cleaved into 16 nonstructural proteins (NSPs) that play important roles in a variety of processes occurring in the virus and host cells [[28], [29], [30]]. Formation of the replicase-transcriptase complex of the virus and rearrangement of membranes of the endoplasmic reticulum into vesicles surrounded by a double membrane, in which replication and transcription of the virus take place, are among these processes [28]. Each of the NSPs has specific function. For instance, NSP1 and NSP2 play important roles in suppression an expression of the host genes, while NSP4 and NSP6 are involved in transmembrane protein formation [16]. The viral genome is transcribed to negative-sense genes of the progeny genome and subgenomic RNA being intermediate products which are used as a templates to synthesis of genomic and subgenomic positive-sense RNAs. The subgenomic RNAs are translated into relevant viral proteins. The structural proteins such as M, S and E are placed in the endoplasmic reticulum-Golgi intermediate compartment (ERGIC) [30]. The newly replicated viral genome forms nucleocapsid with N protein and also moves into ERGIC. There nucleocapsid meets with structural proteins and forms vesicle. After fusion of the vesicle with the plasma membrane, virion is released out of the cell.

## 4 Genome of SARS-CoV-2

**Fig. 3:**
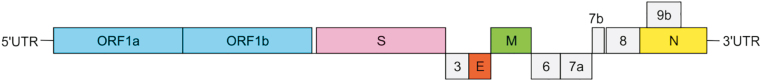
The scheme of the SARS-CoV-2 genome organization. The genome contains 5′-untranslated region (5′-UTR), open reading frame (ORF1a/ORF1b (blue)) that encode non-structural proteins, structural proteins such as spike (S; pink), envelope (E; orange), membrane (M; green) and nucleocapsid (N; yellow), accessory proteins (6, 7a, 7b, 8, 9b; grey), and the 3′-untranslated region (3′-UTR).

The SARS-CoV-2 genome organization was first reported by Wu et al. [31]. The genome exhibits a high similarity to the genomes of previously identified SARS-like coronaviruses [15]. This indicates close relationship of SARS-CoV-2 with these viruses. Indeed, the SARS-CoV-2 RNA genome shares about 96.2% sequence identity with the bat coronavirus RaTG13, while it is in about 79.5% identical to the genome of SARS-CoV-1 [32]. Knowledge about SARS-CoV-2 genome is currently based on the homology to other coronaviruses and on computational predictions. The genome of SARS-CoV-2 is large, and some studies show that it is composed of 14 ORFs, contains 29.811 nucleotides [33], and encodes 9.860 amino acids (AAs) [28,34]. Nucleotide content in the RNA is as follows: 8.903 (29.86%) A, 5.482 (18.39%) C, 5.852 (19.63%) G, and 9.574 (32.12%) U [33]. Two-thirds of the genome constitutes genes encoding non-structural proteins, and the remaining part accounts for structural and accessory proteins [16]. The genome is arranged in a typical 5′–3′ order, and at both ends, it contains untranslated regions (UTR), and, according to some studies, encodes 27 proteins [[29], [35], [36]] (Fig. 3).

ORF1a and ORF1b overlap with a −1 frame shift, encode the pp1a and pp1ab proteins, respectively, and are located at the 5′-terminus. The ORF1ab is the largest gene in the genome of SARS-CoV-2. It contains 15 NSPs (NSP1-NSP10 and NSP12-NSP16). The pp1a protein contains 10 NSPs (NSP1-NSP10) [36]. Some of the SARS-CoV-2 non-structural proteins are already recognized as particular enzymes (Table 1).

**Table 1:** Activity of some SARS-CoV-2 non-structural proteins.

Non-structural protein	Function
NSP3	Papain-like protease (PL^pro^) catalyses cleavage of the SARS-C-V-2 polyproteins, essential for the viral replicase complex formation.
NSP5	Main protease (M^pro^) or chymotrypsin-like protease (3CL^pro^) is responsible for processing the viral polyproteins.
NSP12	RNA dependent RNA polymerase (RdRp) plays the pivotal role in the replication and transcription of the SARS-CoV-3 genome.
NSP13	RNA helicase which possesses also the activity of nucleoside triphosphate hydrolase (NTPase).
NSP14	3' to 5' exonuclease is responsible for proof-reading in the RNA replication. Due to such activity SARS-CoV-2 has a relatively low rate of mutation.
NSP15	Endoribonuclease
NSP16	(Nucleoside-2'-*O*)-methyltransferase (2'-*O*-MTase)

At the 3′-terminus four genes encoding the essential structural proteins, S, E, M and N, as well as genes accounting for accessory proteins are located. The genes that encode the accessory proteins are placed among the structural ones. For SARS-CoV-2 six accessory proteins are currently predicted, yet not all of them are experimentally confirmed [37,38]. In spite of the high homology between genomes of different coronaviruses, they vary particularly in the repertoire of accessory proteins. Therefore, it is very important to characterize the proteins specific for SARS-CoV-2. Recently performed studies with ribosome profiling revealed the translation of over 20 novel viral ORFs. Their significance was not determined; however, they were suggested to play the role of accessory proteins or units able to control synthesis of various viral proteins [39].

## 5 Major druggable targets in SARS-CoV-2

Various studies indicated numerous potential targets associated with SARS-CoV-2 main structural proteins, and enzymes implicated in the key steps of its life cycle. Among these targets Spike protein, papain-like protease (PL^pro^) and chymotrypsin-like protease (3CL^pro^) seem to be particularly important. The three proteins are involved in the virus inoculation and replication in human cells, occur only within the virus, and therefore attract special attention as a possible inhibition site. Catalytic sites of these two proteases and two additional enzymes expressed by SARS-CoV-2, RNA-dependent RNA polymerase and helicase are highly conserved [28]. Therefore, these enzymes, along with the virus main structural proteins, seem to be attractive targets for developing therapeutic strategies and designing promising antiviral drugs.

### 5.1 3CL^pro^

3CL^pro^ (named also main protease (M^pro^)), along with PL^pro^, catalyses cleavage of the two overlapping polyproteins, resulted from the viral RNA translation, into functional NSPs, which are engaged in the formation of subgenomic RNAs encoding accessory and structural proteins of SARS-CoV-2 [40]. 3CL^pro^ acts on the 1 ab polyprotein exclusively, cleaving its sequences after a glutamine residue at 11 distinct sites [41,42], and as the enzyme indispensable for the SARS-CoV-2 maturation, 3CL^pro^ is crucial for its life cycle. Inhibiting the activity of 3CL^pro^ would block the viral replication; enzymes such as RNA-dependent RNA polymerase (RdRp) or NSP13 are not able to fully function without previous proteolytic action of 3CL^pro^; therefore, the M^pro^ of SARS-CoV-2 is an ideal target for drugs, which would prevent spreading of the infection by blocking the polyprotein cleavage. The significance of 3CL^pro^ as a potential therapeutic target is all the more important as there are no human proteases which have similar specificity of cleavage [41]. AA sequence in the M^pro^ of SARS-CoV-2 is in about 96% identical to the enzyme sequence of SARS-CoV-1. These proteases differ in only 12 out of 303 positions in the AAs sequence [43]. Superimpositioned crystal structures of SARS-CoV-2 and SARS-CoV-1 M^pro^ showed conservation of the enzymes active site [42]. M^pro^ of SARS-CoV-2 contains the cysteine and histidine catalytic residues in its active centre. As a monomer, M^pro^ is basically inactive, and undergoes autocleavage [44] before processing the overlapping polyproteins. In the resulted homodimer both protomers are almost orthogonally oriented to each other [45]. They consist of three domains and are bound to each other through *N*-terminal finger situated between the II and III domains. This finger is engaged in the formation of the substrate-binding site situated in a cleft between the I and II domains [41,46]. The residues 8–101, 102–184 and 201–306 are parts of the domains I, II and III respectively. The residues 185–200 constitute a loop which connects domains II and III [46,47]. Two mutations Thr285Ala and Ile286Leu identified in SARS-CoV-2 M^pro^ seem to be behind the higher activity of the enzyme compared to M^pro^ of SARS-CoV-1 [41]. Interesting topological feature of SARS-CoV-2 M^pro^ was recently found by Estrada [43]. It reflects the enzyme capacity to transmission of perturbations across its domains through interactions of the long-range. AAs placed around the binding site near the protease catalytic site show increased sensitivity to such perturbations [43]. It is believed that this finding may be of importance for designing the effective inhibitors of SARS-CoV-2 M^pro^.

### 5.2 S protein

In the case of SARS-CoV-2, as with other enveloped viruses, membrane fusion is a key step in penetration into the host cells. The fusion protein of SARS-CoV-2 is a glycosylated membrane Spike protein, tied up to the virus surface membrane [48]. Fusion of the SARS-CoV-2 and host cell membranes initiates infection. It was found that binding of the SARS-CoV-2 Spike protein to the ACE2 receptor is significantly stronger than the binding of the SARS-CoV-1 analogous protein [49]. During the fusion, the host proteases cleavage the Spike protein resulting in the releasing of its peptide. The process occurs between the S1 and S2 units of the S protein and enable entry of SARS-CoV-2 into the target cell [50]. The SARS-CoV-2 S protein has a sequence of basic AAs which is recognized and cleaved by furin [51]. S protein is formed as a precursor which trimerizes, and it appears as a trimer in mature viruses. The trimer has three receptor binding S1 heads located at the top of the stem of the S2 fusion membrane [52]. The protein is built from S1, S2, and S2' subunits [34]. Through the interactions with the human ACE2 receptor, the subunit S1 contributes to binding of SARS-CoV-2 to the host cell membrane [53]. Six residues of the S protein receptor binding domain (Leu 455, Phe 486, Gln 493, Ser 494, Asn 501, and Tyr 505) were found to be critical for attachment to ACE2. During the process, S protein changes its conformation [24]. The changes are source of the energy needed to overcome the high kinetic barrier of membrane fusion process [48]. Conformational changes of S protein involve the transition from its initial prefusion conformational state to the stable postfusion conformation. Recently, cryogenic electron microscopy structures of the full length SARS-CoV-2 S protein as its prefusion and postfusion conformations were reported, and it was found that transition to the postfusion state is spontaneous and occurs independently of the target cells. It was also observed that the postfusion structure possesses N-linked glycans which may be indicative of a protective role against the host immune response [48]. The subunit S2 is involved in fusion of the viral membrane and host cellular membrane. During the fusion, the S2 subunit adopts three main conformational states: prefusion native state, prehairpin intermediate state, and ensuing postfusion hairpin state [34]. It was discovered that in the S protein of SARS-CoV-2 four AA residues were placed between the subunits S1 and S2, and that their presence affects the S protein cleavage [50]. Consequently, S protein shows the unique furin cleavage site is located within the S1/S2 domain between the residues 682 and 685, and overlaps with the insertion [50]. Lately it was shown that the receptor binding domain of SARS-CoV-2 S protein is able to bind linoleic acid in three binding pockets [54]. This stabilizes a locked S protein conformation and reduces ACE2 interactions in vitro. Synergistic effect of remdesivir supplemented with linoleic acid on suppression of SARS-CoV-2 replication was also demonstrated. In human epithelial cells infected with SARS-CoV-2, addition of linoleic acid markedly reduced the dose of remdesivir necessary to inhibit viral replication [54]. This suggests that the linoleic acid-binding pocket within S protein of SARS-CoV-2 constitutes a promising target for potential drugs.

It is worth noticing that mutations within the S protein can have serious consequences. For example, the D614G mutation makes virions more stable and infectious, which has a positive effect on SARS-CoV-2 replication and leads to the virus spread [55,56].

### 5.3 RdRp

RdRp is the enzyme playing the pivotal role in the replication and transcription of the SARS-CoV-2 genome. Replication-transcription complex, consisting of viral non-structural proteins, takes part in these processes. NSP12, the catalytic subunit of RdRp, constitutes core protein of the complex [57]. For the proper functioning of the enzyme, accessory subunits NSP7 and NSP8 are required [57]. The core protein contains a single chain of approximately 900 AAs [58], N-terminal nidovirus RdRp-associated nucleotidyltransferase domain, interface domain and C-terminal RdRp domain [59,60]. The RdRp domain adopts the conserved architecture of the viral polymerase family, looks like a right hand and comprises three subdomains: fingers, palm and thumb. NSP7 and NSP8 are attached to the thumb, and copy of the NSP8 subunit forms bond with the fingers domain [59,61]. Generally, the architecture of the SARS-CoV-2 replication-transcription complex resembles that of SARS-CoV-1; in spite of this, some differences between them are observed. The portion of the nucleotidyltransferase domain in SARS-CoV-1 is composed of six helices with a three-stranded β sheet at the N terminus, while in SARS-CoV-2 a structural block with five antiparallel β strands and two helices is present [59]. Conserved motifs of the palm subdomain form the active site of the SARS-CoV-2 RdRp domain [59]. The active site is not only the most conserved, but also relatively easily accessible region. Therefore, inhibiting viral replication by blocking the RdRp active site can be an effective therapeutic approach.

### 5.4 PL^pro^

The PL^pro^ is, along with 3CL^pro^, an enzyme essential for the viral replicase complex formation thereby contributing to its spread [62]. PL^pro^ is also involved in the cleavage of post-translational modifications of the host proteins. This action is perceived as a mechanism to avoid the host's antiviral immune response [62,63]. In the enzyme active site three residues: Cys112–His 273–Asp 287, which are located at the interface of the subdomains thumb and palm, form the catalytic triad [64]. The action of PL^pro^ is based on the catalytic cycle of cysteine protease. In this cycle, Cys112 acts as a nucleophile, His273 acts as an acid-base, and Asp287 paired with histidine promotes Cys112 deprotonation [64]. Although PL^pro^ of SARS-CoV-2 and PL^pro^ of SARS-CoV-1 share 83% sequence identity, the enzymes exhibit different host substrate preferences; the SARS-CoV-2 protease preferentially cleaves the ubiquitin-like protein ISG15 (regulator of host innate immune pathways), while PL^pro^ of SARS-CoV-1 predominantly targets ubiquitin chains [62]. Taking into consideration the functions performed by PL^pro^, it seems to be clear that its inhibition can lead to dual therapeutic effect: suppression of SARS-CoV-2 infection and promotion of anti-viral immunity.

### 5.5 E protein

E protein is involved in virus pathogenesis, assembly, and release, playing in these processes multifunctional roles [65]. Among the virus structural proteins, E protein that has in its sequence about 75 AAs, is the smallest. E proteins, from different coronaviruses, share common general structure. The structure is characterized by the presence of amino-terminal, transmembrane and C terminal domains [65,66]. The first of the three domains is short and has hydrophilic character; the second one is large and hydrophobic. The protein functions also as an ion-channelling viroporin. Although the sequence of AAs in E protein can vary among different viruses, it is very similar in SARS-CoV-2 and SARS-CoV-1. As the protein is involved in some critical aspects of the viral life cycle, E protein seems to be a good drug target.

### 5.6 M protein

M protein is small, about 222 AAs long, structural protein of SARS-CoV-2. It maintains the virion shape. This protein is characterized by the presence of three transmembrane domains. Other features of M protein are: N-terminal and C-terminal domains. The first domain is short, glycosylated and located outside the virion; the second domain is larger and situated in the virion. It was suggested that dimer is the active form of M protein in virion. Membrane protein is able to adopt different conformations, thus enabling promotion of membrane curvature but also binding the nucleocapsid [65,67].

### 5.7 N protein

N protein plays multiple functions which are crucial for the viral life cycle. Therefore, the N protein is a very important antiviral drug target. This is highly conserved and the only structural protein which through direct binding to viral RNA induces its stability. The N protein is involved in various significant processes such as ribonucleoprotein formation during viral RNA genome packaging, control of RNA synthesis during replication and transcription, and modulation of the metabolism of infected cells. In all these processes, the N protein plays many important roles. During infection, this very immunogenic protein is highly expressed [68].

The protein contains an N-terminal domain and a C-terminal domain. Between them there is an RNA-binding domain or a linker region (LKR) [65,69]. The crystal structure of the N-terminal domain of SARS-CoV-2 N protein reveals specific distributions of the surface charge. This finding may facilitate discovery of the SARS-CoV-2 dedicated drug [70].

### 5.8 Helicase

NSP13 of SARS-CoV-2 was found to possess the activity of both nucleoside triphosphate hydrolase (NTPase) and RNA helicase [71]. The protein is able to hydrolyse all kinds of NTPs (with a preference for ATP and GTP), and unwind RNA helices dependently of the presence of NTP. The SARS-CoV-2 NSP13 activity as an NTPase is manifested in the presence of a divalent metal ion. NSP13, as a helicase, unfolds the RNA helix towards 5'-3', in the presence of ATP and Mg^2+^. This activity is consistent with that of the helicases of other RNA viruses [71].

Helicase of SARS-CoV-2 which structure was elucidated by computational studies [72] is thought to have 596 AAs which are placed in pplab from 5325 to 5925 position. The enzyme has five domains which adopt the structure of a triangular pyramid shape. Three of these domains: 1A (261–441 AAs), 2A (442–596 AAs), and 1B (150–260 AAs) form the triangular base. N-terminal zinc binding domain (1–99 AAs) along with stalk domain (100–149 AAs), serving as the junction between the ZBD and 1B domains, is at the pyramid apex. Active site of the SARS-CoV-2 helicase contains the following residues: Lys 288, Ser 289, Asp 374, Glu 375, Gln 404 and Arg 567. The residues are placed together in the cleft at the base between the domains 1A and 2A. Due to the high similarity of the AA sequences of NSP13 proteins from different coronaviruses, the elucidated structure can be considered the most likely.

RNA helicases, which exhibit the activity of NTPase and catalyze breaking of the hydrogen bonds between base pairs using the energy of ATP hydrolysis, play an important role in all processes of the viral life cycle, in which RNA is involved. Therefore, SARS-CoV-2 helicase can be perceived as the potential target for antiviral therapy development.

## 6 Accessory proteins

The SARS-CoV-2 accessory proteins have recently attracted some attention from the scientific world. This is most likely due to the growing evidence that in spite of being not directly involved in the SARS-CoV-2 replication, these proteins play various essential roles in the virus pathological processes [[73], [74], [75], [76]]. Moreover, while the organization of genomes among members of the *Coronaviridae* family is similar in terms of structural and non-structural proteins, clear differences are present with respect to the number, location and sequence of genes encoding accessory proteins [77,78]. All these mean that in addition to structural and non-structural proteins, the accessory ones may represent valuable therapeutic targets. Among the nine accessory proteins encoded by the SARS-CoV-2 genome [79], i.e., ORF3a, 3b, 6, 7a, 7b, 8, 9a, 9b and 10, two proteins, ORF3a and ORF8, seem to be particularly important.

ORF3a is the largest from the accessory proteins. It is a viroporin protein forming ion channels in the membrane of the host cell [80]. The protein structure contains three transmembrane helices and a cytosolic domain. The ability to form the channels results from a dimeric form of ORF3a. The channels are formed by the six dimer helices with their residues placed inside. Thus the channels are capable of cations conducting, preferentially Ca^2+^ and K^+^ [80]. The ORF3a protein blocks mechanism of autophagy [76], causes damage of lysosomes resulting in their improper functioning [76], and is implicated in the inflammasomes activation which can influence the cytokine storm development. ORT3a is also involved in cell apoptosis [73], and it was found that mutations within this protein are responsible for a higher rate of mortality caused by SARS-CoV-2 [81].

ORF8 contains 121 AAs, and is the second-largest accessory protein encoded by the genome of SARS-CoV-2 [75]. The main components of the protein structure are the sequence of N-terminal signal for import to endoplasmic reticulum and a core of β-strand which is similar to the (Ig)-like fold of immunoglobulin [75,80]. ORF8 does not contain the C terminal transmembrane domain. The protein induces the strongest and very specific immune system response; therefore, its antibodies are among the primary markers of the infection caused by SARS-CoV-2 [82]. ORF8 was also found to act as an antagonist of the interferon (type I) [83]. ORF8 is characterized by high variability of structure and tendency to undergo mutations [84].

ORF6 is also involved in the modulation of the host innate immune system. Similar to ORF8, ORF6 was found to be an antagonist of interferon. This protein inhibits the interferon synthesis by perturbing the upstream as well as the downstream signaling pathway [83]. It was shown that the ORF6 C-terminus is essential to the inhibitory activity exerted by this protein [83].

ORF7a is a transmembrane protein [80] capable of interacting with the ribosomal transporting proteins. Moreover, protein activity possibly leads to the inhibition of cellular translation [85].

ORF10 is unique among all the proteins encoded by the SARS-CoV-2 genome because it contains an AA sequence that does not resemble that of any other known protein [86]. This caused difficulties in the prediction of the protein role in the virus pathogenesis. Although some functions of the hypothetical protein were speculated, the recent studies indicate that ORF10 does not affect the course of COVID-19, or the infectivity of SARS-CoV-2, and seems to be inessential [86].

## 7 Human enzymes implicated in SARS-CoV-2

**Fig. 4:**
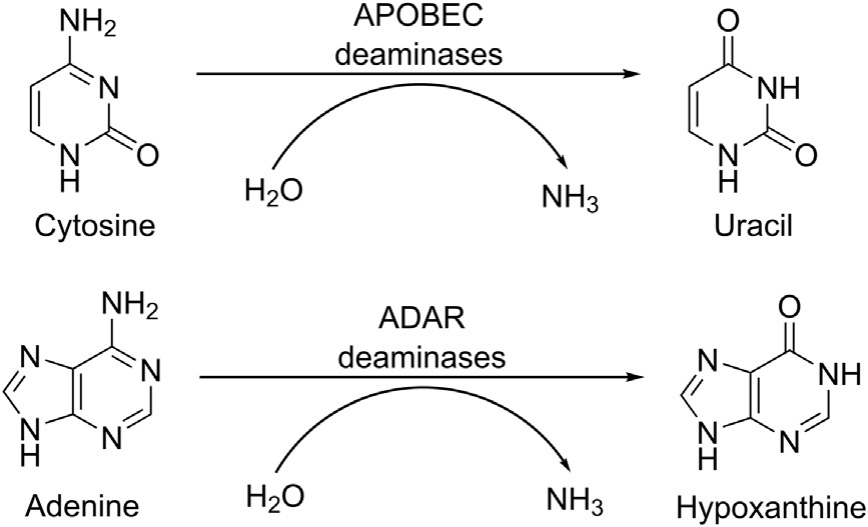
Deamination of cytosine and adenine. APOBEC: apolipoprotein B mRNA editing catalytic polypeptide-like, family of cytidine deaminases; ADAR: adenosine deaminase that acts on RNA or adenosine deaminase RNA specific.

In human cells, two groups of proteins ADAR and APOBEC are involved in the editing of RNA [87]. ADARs deaminate adenosine to inosine which, in the process of translation and splicing, is recognized as guanosine [88,89], while APOBEC is responsible for deamination of cytidine to uridine [90] (Fig. 4).

When SARS-CoV-2 infects the human cells, enzymes from the both families may potentially act on the viral RNA in a similar way to their action on the host RNA. In the genome of SARS-CoV-2, the content of the cytidine (C) is very low (18.39%), while the uridine (U) content is high (32.12%) [91]. Earlier it was shown that genomes of several other *Coronaviridae* members, among them of those infecting humans, exhibit the similar property [92]. Among the coronaviruses, HCoV-NL63, HCoV-OC43, BCoV, IBV, HCoV-229E, TGEV, MHV, PEDV and SARS-CoV-1, the genome of HCoV-NL63 was characterized by the lowest GC content equal to 34%, and by a very high content of U (39%) compared to only 14% of C [92]. Moreover, genomes with the lowest amount of C (HCoV-NL63, HCoV-OC43 and BCoV) were found to have the highest U content and vice versa [92]. Indeed, taking into account all these results together with the nucleotides content in the genome of SARS-CoV-2, it seems clear that the composition of nucleotides in the genomic positive RNA strand of various representatives of *Coronaviridae* family has a common feature: U is the most abundant nucleotide while G and particularly C are underrepresented.

Recently, RNA obtained from bronchoalveolar fluids from patients infected with SARS-CoV-2 were analyzed and nucleotides changes that might be indicative of the host deaminases involvement in the RNA editing were identified [93]. Changes of adenosine to inosine and of cytosine to uracil were observed [93]. This finding suggests that the both groups of proteins APOBEC and ADAR are involved in the SARS-CoV-2 genome editing. This fact can be of great importance with regard to development of new therapies for treatment of COVID-19. We suggest that it might be beneficial to increasing the involvement of both deaminases but particularly of APOBEC in editing of the virus RNA. This might lead to upregulation of changes in the genomic RNA, and consequently to a loss of genome integrity.

## 8 Novel usage of known agents

No specific drugs are currently available for treatment of COVID-19; however, various existing pharmaceuticals originally designed to combat other diseases are tested worldwide for their potential activities against the novel coronavirus. Also, diverse natural products, known for their wide spectrum of biological activities including antiviral properties, can be used against SARS-CoV-2. Candidate drugs, belonging to various categories, possibly acting on different stages of the virus life cycle against different viral targets including structural but also non-structural proteins, are comprehensively reviewed in this section. Diverse approaches to therapeutic strategies are also briefly discussed.

### 8.1 Potential drugs for COVID-19

#### 8.1.1 Medicaments blocking virus membrane – host cell membrane fusion

##### 8.1.1.1 Hydroxychloroquine and azithromycin

**Fig. 5:**
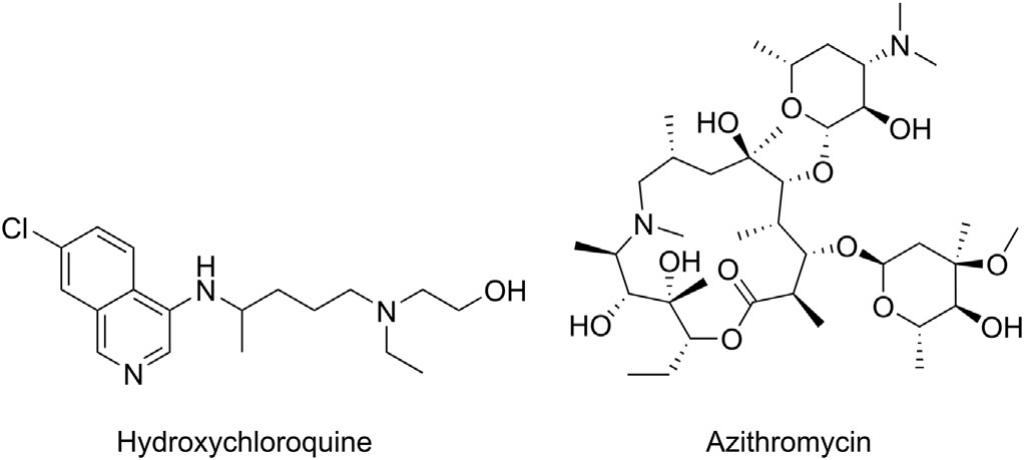
Structures of hydroxychloroquine and azithromycin.

Hydroxychloroquine and azithromycin (Fig. 5) are antimalarial and antibacterial drugs currently used for the COVOD-19 treatment [[94], [95], [96]]. Hydroxychloroquine exhibits also anti-inflammatory and antiviral properties, and for this reason, studies of its effectiveness as anti- SARS-CoV-2 agent were undertaken. The drug acts as a weak base, and therefore is able to affect pH and thus inhibit diverse enzymes [97]. Through inhibition of the ACE2 glycosylation, it can block SARS-CoV-2 entry to the cell [94,97,98] due to possibly less efficient interaction between the unglycosylated receptor and the virus S protein [97]. It can block the viral post-translational modifications, and affects on protein degradation in the lysosome [95]. It is also suggested that hydroxychloroquine can block the virus maturation resulting in failure of further virions transport [98].

Hydroxychloroquine is being used for treatment of COVID-19 in most of the countries but the curative effects observed are not unequivocal. Some reports suggest that treatment with hydroxychloroquine does not reduce the risk of the appearance of most serious sequelae of the disease [99,100]. The other study shows a higher mortality rate in the patients treated with this drug but at the same time points out the reduced risk of their mechanical ventilation.

Although numerous ways of activity lying at the basis of antiviral properties showing by azithromycin were proposed, the exact mechanism behind the therapeutic effect caused by the drug against SARS-CoV-2 remains unknown. It is suggested that similar to hydroxychloroquine, azithromycin acts as a weak base increasing pH of endosome maturation and trans-Golgi network [95,101]. If so, the drug acts directly on the cells of the bronchial epithelium and reduces the secretion of mucus, thereby improving the functioning of the lungs [95]. It is supposed that through interactions between the S protein and ACE2 receptor, azithromycin interferes with the virus entry, resulting in blocking this process [95].

##### 8.1.1.2 Umifenovir (Arbidol)

**Fig. 6:**
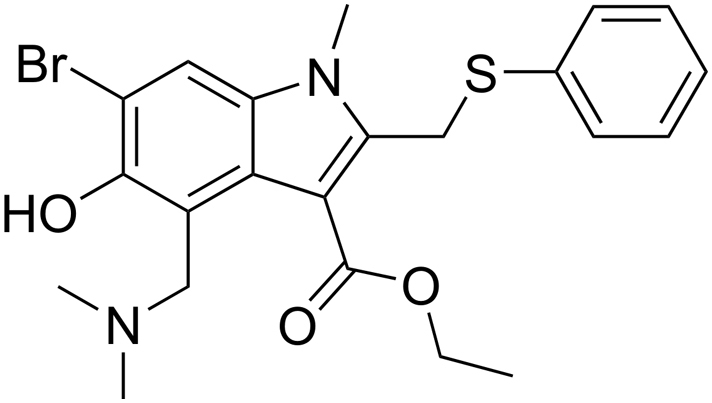
Structure of umifenovir.

Umifenovir (Fig. 6), heterocyclic compound containing an indole core functionalized with different substituents, is an antiviral agent which is reported to obstruct fusion of viral and the host cell membranes, and therefore is claimed to inhibit viral entry into the target cells [102]. Incorporated into the cell membranes, umifenovir interferes with the network of hydrogen bonds of phospholipids [103,104]. The drug obtained the license for influenza treatment in China and Russia. In the case of influenza virus, the drug stabilizes hemagglutinin through direct interactions with the viral particles and reduction of probability of reaching low pH essential for the conformational transition of the enzyme into its functional fusogenic form [105]. However, data concerning umifenovir efficacy as an anti-SARS-CoV-2 agent are controversial. For example, the drug was proposed to be an efficient inhibitor of SARS-CoV-2 in vitro [106], although another study demonstrated that treatment of patients with COVID-19 with this drug is not associated with expected results [107]. Overall, there are no strong data to support the use of umifenovir to improve outcomes in COVID-19 patients [102].

#### 8.1.2 Inhibitors of RdRp: favipiravir and remdesivir

**Fig. 7:**
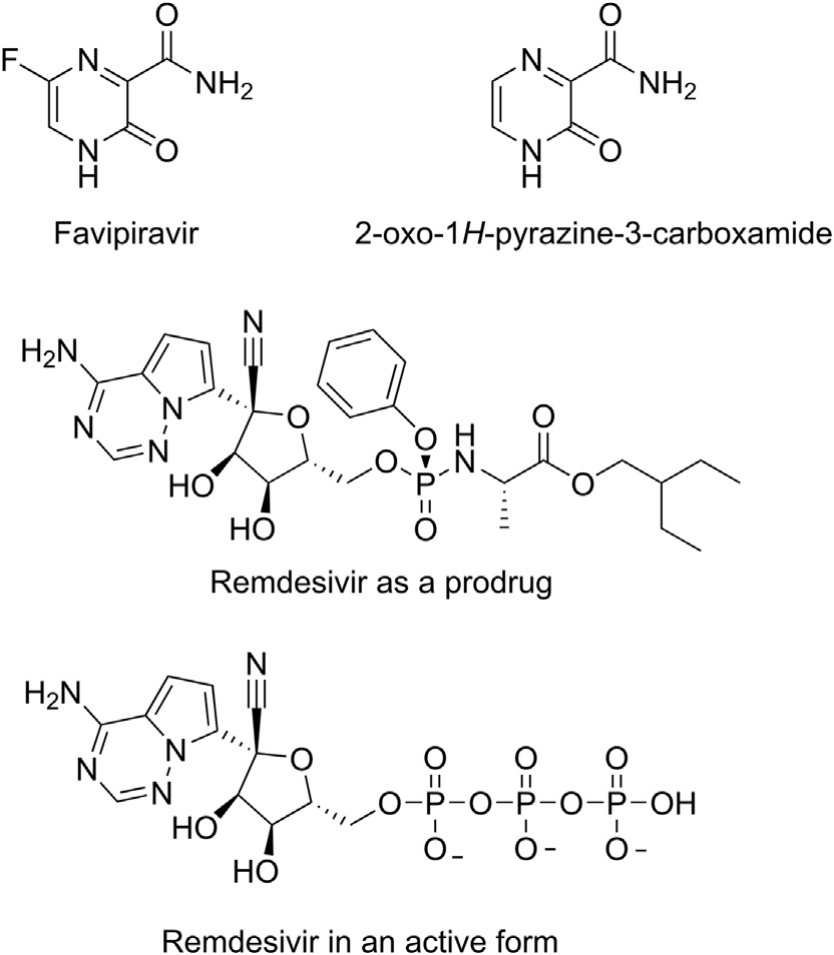
Structures of favipiravir, 2-oxo-1*H*-pyrazine-3-carboxamide, and remdesivir as a prodrug and its pharmacologically active form.

Favipiravir and remdesivir (Fig. 7) are antiviral agents acting as RdRp inhibitors [97,108], the enzyme is involved in transcription and replication of the viral genome. Favipiravir selectively and strongly inhibits this enzyme in numerous RNA viruses, including a wide range of influenza viruses, Ebola and Lassa viruses [97,108].

Due to the positive effect that favipiravir has on RNA viruses, it is considered to be used to treat patients with COVID-19, especially in that the drug was found to be active against the causative agent of the disease, SARS-CoV-2, in vitro. Also, preliminary clinical results showed health benefits for patients treated with favipiravir with regard to control group of patients to whom another drug was administered [109]. Favipiravir is currently in clinical trials. The first two phases of the research ended with promising results [97]. Recently, in silico studies focused on identification of favipiravir structural analogues potentially active against SARS-CoV-2 were performed, and 2-oxo-1*H*-pyrazine-3-carboxamide (Fig. 7) was concluded to be an excellent drug candidate which can be more effective than favipiravir [108]. It is worth noticing that favipiravir differs only from its chosen analogue in a fluorine atom attached to the heterocyclic ring.

Remdesivir (Fig. 7) is a 1̍-cyano-substituted derivative of adenosine C-nucleoside phosphoramidate and a prodrug whose pharmacologically active form is a nucleoside triphosphate analogue. Remdesivir is metabolized to the active form before it enters human respiratory epithelial cells [110,111]. As an adenosine analogue, remdesivir competes with the cellular adenosine triphosphate, thus inhibiting the viral RdRp. The ability to function by effective competing with naturally occurring ATP is a unique feature of remdesivir [112]. While incorporating into the strand of forming RNA, the drug stops the replication of the virus [110,113]. It blocks the enzyme while reaching the third position from its active site, and inhibits the RdRp from inclusion of the next nucleotide [110,112]. Selectivity over cellular adenosine triphosphate and delayed chain termination at position *i*+3 are key steps in the mechanism responsible for RdRp inhibition by remdesivir [112]. On the basis of results obtained with regard to inhibition mechanism, remdesivir was defined as a direct-acting antiviral agent [112]. Remdesivir was designed to treat Ebola virus disease; however, the drug exhibits a wide spectrum of activities against RNA viruses including MERS and SARS-CoV-1 [[114], [115], [116], [117]]. Recently, it was shown that the drug has a moderate clinical efficacy against SARS-CoV-2 [118]. The data concerning the human safety, and results of studies with cell culture and animal models seem to justify the ongoing clinical trials for evaluating the efficacy of remdesivir in hospitalized patients with COVID-19.

#### 8.1.3 Chemical agent acting on the immune system: dexamethasone

**Fig. 8:**
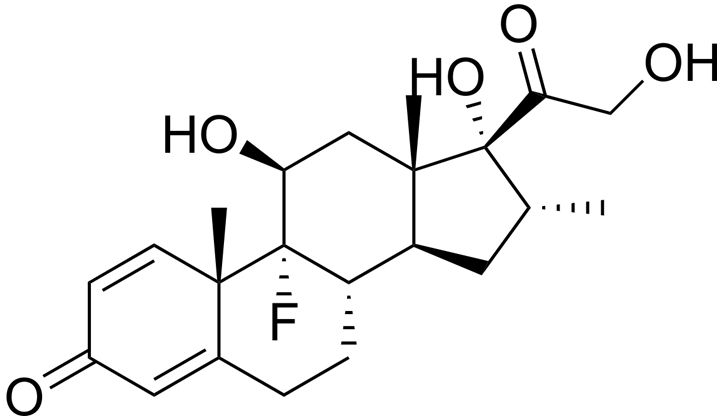
Structure of dexamethasone.

Dexamethasone (Fig. 8) is a synthetic corticosteroid with powerful anti-inflammatory activities. Due to the presence of fluorine atom in its structure, the compound exhibits glucocorticoid effects. The administration of low and high doses of glucocorticoids leads to an increase in the production of anti-inflammatory compounds, while high doses reduce the secretion of pro-inflammatory compounds, including cytokines, chemokines and pro-inflammatory enzymes such as phospholipase A2 and cyclooxygenase. The mechanism of action of dexamethasone against SARS-CoV-2 is not clear [97]; however, since COVID-19 is associated with lung injury, the drug appears to be able to affect the lung damage caused by inflammation and thus reduce progression to respiratory failure and death. Nevertheless, using of dexamethasone during the early phase of the viral infection might result in increased viral replication and aggravate the disease. Currently, dexamethasone is used in the treatment of hospitalized patients with COVID-19 in the United Kingdom. A preliminary report shows that the use of dexamethasone led to lower mortality among patients who required mechanical ventilation or received oxygen, but not among those who did not receive respiratory support [119].

#### 8.1.4 Plasma and antibody therapies

Plasma therapy was earlier used in the SARS and MERS outbreaks, and at present it is found to also have good efficacy for patients with critical COVID-19 [120]. Studies show that convalescent plasma with neutralizing antibodies derived from recently recovered donors, was well tolerated and increased or maintained the neutralizing antibodies at a high level [121].

The neutralizing monoclonal antibodies against SARS-CoV-2 can be used therapeutically and prophylactically. Their main target is S glycoprotein. Most isolated monoclonal antibodies are directed at the binding domain which enables SARS-CoV-2 to contact the ACE2 receptor. However, it seems that neutralizing antibodies can also be used against other regions in the S protein [122]. Several SARS-CoV-2 monoclonal antibodies are planned to enter clinical trials. Given the relatively long half-life of most monoclonal antibodies, it appears that a single infusion should be sufficient for obtaining health benefits.

### 8.2 Therapeutic nucleotides

Other strategies which can be used against SARS-CoV-2 are based on nucleic acid technologies and comprise using of short interfering RNAs (siRNAs), antisense oligonucleotides (ASOs), and also ribozymes. Several such technologies were already studied and used against respiratory viruses: influenza, measles, respiratory syncytial virus and models of SARS-CoV-1 [123].

siRNAs are double-stranded RNA usually about 20 nucleotide long. They cause the degradation of the specific genes mRNA with complementary nucleotide sequences, and thereby inhibit their expressions and prevent translation [124]. A few siRNAs about potential antiviral activity were prepared to target different sites within the genome of SARS-CoV-1 [125]. In studies in which the cultures of Vero cell and animal models were used, some of the siRNAs caused a decrease in replication of the virus and weakness of the cytopathic effects [126]. This demonstrates usefulness of synthetic siRNAs in the fight against the virus. The siRNA used against the sequence of S protein also showed the significant inhibitory effect [127].

Ribozymes are RNA molecules showing catalytic activity. They occur in nature and their main natural function is to cleave phosphodiester bonds within the nucleic acids [125]. Among different groups of ribozymes, only the hammerhead and hairpin ones received particular attention due to their small size. In different studies, the hammerhead and hairpin disrupted and reduced viral replication and thus effectively inhibited pre-genomic RNA levels of infecting viruses [125]. A chimeric DNA–RNA hammerhead reduced the expression of SARS-CoV-1 RNA in 3T3 cells transfected with the recombinant plasmid [128].

Antisense oligonucleotides are small, containing usually 15–30 nucleotides in length, synthetic pieces of single-stranded DNA. They act by the creation of double-stranded structures through formation of the Watson-Crick hydrogen bonds with the target complementary DNA or RNA sequences. Involved in the bonding with target RNA, they induce its cleavage or inhibit maturation of mRNA. This may result in the inhibition of the translation processes [129]. Studies performed with modified ASOs used against different respiratory viruses showed promising results. Specific three peptide-conjugated phosphorodiamidate morpholino oligomers were designed against specific sequences in the SARS-CoV-1 genome [130]. Studies performed with the ASOs suggested their therapeutic potential against the virus infection [131].

Large genome of SARS-CoV-2 potentially offers numerous targets for therapies based on nucleic acid technologies. Combinatorial approach to the COVID-19 problem was suggested [16]. This approach indictates application of technology wherein a cocktail of multiple oligonucleotides against the key region of the SARS-CoV-2 genome is used. Such a strategy increases the chance of obtaining a satisfactory therapeutic effect. A similar approach is already used in the fight against HIV [16]; however, its application as a strategy against SARS-CoV-2 would require taking into account the biology of this virus completely different from that of the retrovirus.

The biggest difficulty in the use of technologies based on nucleic acids is associated with the delivery of therapeutic nucleotides to target cells in vivo. The question is how the nucleotides could be introduced into the lungs, and how they could be delivered in amounts sufficient to stop the infection or at least alter its clinical course, and provide clear health benefits. In the body fluids, nucleic acids undergo enzymatic digestion. They have a highly charged nature which makes it impossible to cross the cell membrane in the free diffusion process. These properties, besides nucleic acids immunogenicity and phagocytosis, constitute the major challenges for the delivery of such kind of potential therapeutics to the target cells [123]. With respect to respiratory viruses, siRNA was reported to be administered by the use of liposomes, polymers or other cationic systems [123].

Reaching of the target cells, stability and expression of the therapeutic nucleotides are the most important aspects that influence the efficacy of strategies based on nucleic acid technologies. Despite all the difficulties associated with their delivery to a specific target, properly designed nucleic acids can exert a significant inhibitory effect and are certainly worth considering as potential agents against SARS-CoV-2. Application of a few therapeutic nucleotides may result in a synergistic effect against the viral propagation. However, there is still a rather limited ability to prepare the drugs based on the nucleotides to a larger group of infected people [132].

### 8.3 Natural products as potential anti-SARS-CoV-2 agents

Natural products along with the synthetic compounds inspired by the natural products structure remain a valuable source for discovery of new drugs, including therapeutics about antiviral activity. Natural products of both plant and microbial origins are good basis for new drug development; the uniqueness of compounds derived from microbial sources is due to their chemical diversity [133], but extracts from plants also contain a wealth of remarkable bioactive metabolites [134]. Searching for therapeutic agents which would be selective towards SARS-CoV-2 and would show low toxicity for the host cells turned the scientific world's attention to natural products with antiviral activities. Earlier, numerous compounds of various flora and fauna origins were evaluated in vitro tests for activities against SARS-CoV-1. The effects of over 200 specific phytocompounds on SARS-CoV-1 using a Vero E6 cell-based cytopathogenic effect assay were evaluated [135], and some of these compounds were demonstrated to exhibit remarkable activities reflected in low IC_50_ values (Table 2). These compounds belong to terpenoids (Table 2, compounds 1**–**4 are diterpenoids of abietane-type, compound 5 is a representative of triterpenoids of lupane-type), and lignoids (compound 6). These results show that indeed, natural metabolites have a great potential as biologically active substances, and can provide a new direction for development of anti-SARS-CoV-1 and possibly also anti-SARS-CoV-2 agents.

**Table 2:** Chemical structures of the compounds that exhibit significant inhibitory activity against the cytopathogenic effect of SARS-CoV-1 on Vero E6 cells along with the IC_50_ values [135].

No.	Compound name	Compound structure	IC_50_ (μM)
1	Ferruginol		1.39
2	8β-Hydroxyabieta-9 (11),13-dien-12-one		1.47
3	7β*-*Hydroxydeoxycryptojaponol		1.15
4	3β,12-Diacetoxyabieta-6,8,11,13-tetraene		1.57
5	Betulonic acid		0.63
6	Savinin		1.13

**Fig. 9:**
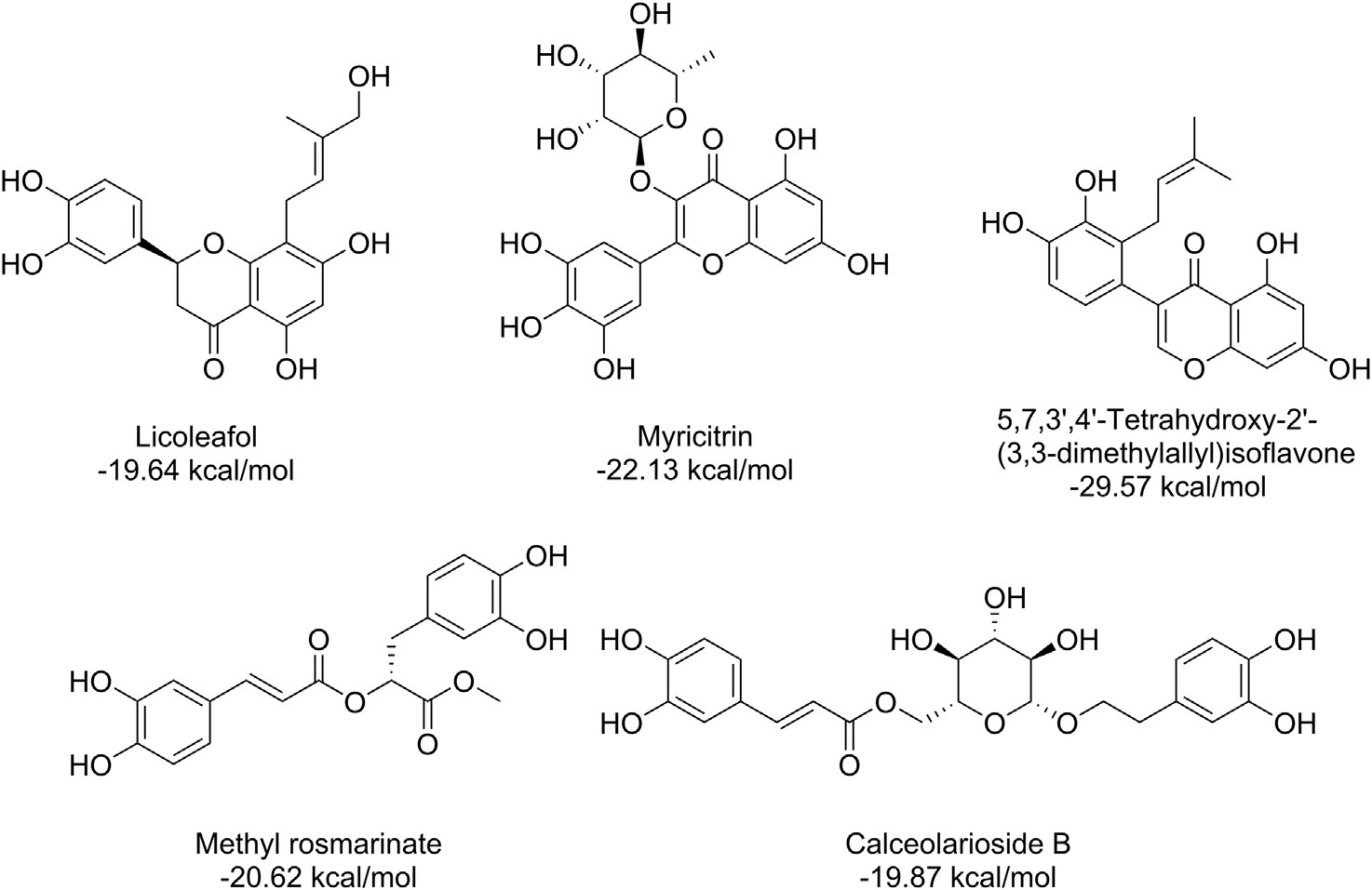
Phytochemicals against SARS-CoV-2 M^pr^^o^ receptor binding site, with their respective structures and binding affinities [46].

Numerous molecules belonging to different types of compounds such as anti-tussive, anti-influenza, glucosinolates, flavonoids, terpenes, terpenoids and alkaloids along with synthetic anti-viral molecules as references compounds were analyzed [136]. Several natural molecules were identified to bind strongly to M^pro^ of SARS-CoV-2. Interestingly, these molecules also showed strong affinities for other potential therapeutic targets associated with the infection caused by SARS-CoV-2 such as viral RdRp and human ACE2 [136]. The best results of docking analysis obtained for selected natural molecules and remdesivir as a reference anti-viral agent are presented in Table 3. The flavonoids show particularly high binding energy with all three targets, suggesting these compounds to be good candidates for further studies aimed at multi-targeted drug development against COVID-19. On the other hand, myricitrin was shown to have binding affinity with the SARS-CoV-2 M^pro^ binding site, equal to −22.13 kcal/mol [46]. In this study a few more molecules were demonstrated to exhibit the binding affinity higher or close to −20.0 kcal/mol (Fig. 9) [46].

**Table 3:** Natural products and their binding energy (kcal/mol) values to M^pro^, RdRp and ACE2 obtained by docking analysis [136].

No.	Compound name	M^pro^	RdRp	ACE2
Anti-tussive agents				
1	Myricitrin	−8.9	−7.9	−7.5
2	Chrysanthemin	−8.8	−7.7	−7.6
3	δ-Viniferin	−8.4	−8.3	−8.4
Alkaloids				
4	(−)-Asperlicin C	−9.7	−8.7	−9.5
5	(+)-Asperlicin E	−8.8	−9.1	−7.7
6	Oriciacridone F	−9.1	−9.6	−6.7
Flavonoids				
7	Cinnamtannin A1	−11.0	−10.8	−8.2
8	Epicatechin-(4β,8)-epicatechin-(4β,6)-catechin	−10.6	−9.9	−8.2
9	Anemone purple anthocyanin 1	−10.4	−10.2	−7.4
10	Anemone blue anthocyanin 3	−10.1	−12.7	−7.7
11	Cinnamtannin D2	−10.0	−8.8	−7.7
12	Anemone blue anthocyanin 1	−10.0	−13.2	−6.8
13	Gambiriin B2	−10.0	−10.4	−8.6
14	Epicatechin-(4β,8)-gallocatechin	−10.0	−10.0	−7.6
15	Cinnamtannin A3	−9.9	−10.4	−6.5
16	Eriodictioside	−9.9	−12.5	−7.4
17	Quercetin 3-glucosyl-(1,4)-rhamnoside	−9.9	−9.8	−6.5
18	Taiwanhomoflavone A	−9.6	−9.8	−7.8
Reference antiviral compound				
19	Remdesivir	−8.2	−7.4	−7.8

Besides flavonoids, volatile terpenoids as well as algae polyphenols can be a source of substances having possible inhibitory activity against different targets in SARS-CoV-2 [134].

#### 8.3.1 Curcumin

**Fig. 10:**
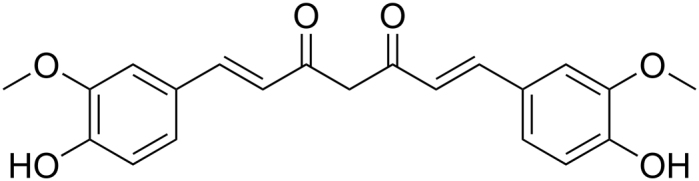
Structure of curcumin.

Curcumin (Fig. 10) is a natural compound isolated mainly from Curcuma longa [137]. This compound has long been known to be used as a natural medicine for treatment of various human diseases.

Curcumin and its analogues were shown to significantly inhibit production of cytokines: IL-1, IL-6, IL-8, TNF-α, which have pro-inflammatory activity [138,139] and are involved in cytokine storm that can, among destruction of various tissues and cell, cause damage to the lung. This can lead to acute respiratory distress syndrome (ARDS) and the failure of multiple organs. Moreover, curcumin was demonstrated to decrease expressions not only of the mentioned cytokines but also other mediators of inflammatory processes, which are involved in the regulation of the immune cells activity and promotion of lung fibrosis [137,138]. Apart from showing inhibitory activity against pro-inflammatory molecules, curcumin can positively influence anti-inflammatory cytokines regulation: it upregulates anti-inflammatory IL-10 [140,141]. As a polyphenolic antioxidant which in its molecule, apart from hydroxyl groups, contains β-diketone moiety, the compound acts also as a reactive oxygen species (ROS) scavenger [137]. It effectively removes ROS and inhibits the lecithin peroxidation. It also inhibits the ROS induced oxidative damage to DNA. Recently, it was reported that curcumin opposes the effect of ROS on the expressions of pro-inflammatory cytokines [142]. This natural medicine is able to disrupt infections caused by various viruses including SARS-CoV-1 [135]. Curcumin was shown to inhibit the activity of the SARS-CoV-1 3CL^pro^, one of the key enzymes for the virus replication. The compound can also inhibit pulmonary fibrosis. All these findings suggest that curcumin may effectively improve inflammation and be used in treatment of viral infections, and can be considered as an attractive supportive agent for COVID-19 treatment.

#### 8.3.2 Resveratrol

**Fig. 11:**
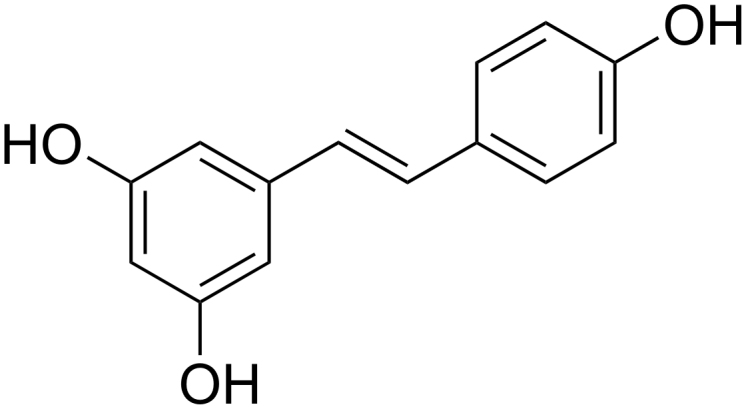
Structure of resveratrol.

Resveratrol (Fig. 11) is a representative of stilbenoids, natural phenolic compounds produced by plants (for example grapes, blueberries, and peanuts) in response to stressful conditions such as ultraviolet radiation, ozone or bacterial and fungal infections [143].

Although, under physiological conditions, resveratrol is poorly soluble, characterized by rapid metabolism and low plasma bioavailability [144], innovative drug delivery systems using liposomes, micelles, emulsions and, above all, lipid nanocarriers, as well as its insertion into polymeric nanoparticles, and nanocrystals enhance pharmacokinetics parameters of the compound [145]. Resveratrol shows cardioprotective, anticancer, antifungal and antimicrobial activities [143]. It has also inhibitory activities against viral replication and virus-induced inflammation in diseases caused by several human pathogens including respiratory viruses [146], and its derivatives were shown to suppress SARS-CoV-1 replication [147]. Recently, in silico studies aimed at evaluating stilbenoid analogue activity against SARS-CoV-2 S protein and human ACE2 receptor were performed [148]. The studies, in which molecular docking was used, showed that resveratrol has binding affinity > −7 kcal/mol. In addition, the application of molecular dynamics simulations taking into account the aquatic environment showed that resveratrol binds to the S protein: ACE2 receptor complex [148]. These data indicate that resveratrol can be considered as a promising drug candidate against COVID-19, capable of interfering with the activity of the virus S protein.

#### 8.3.3 Cannabidiol

**Fig. 12:**
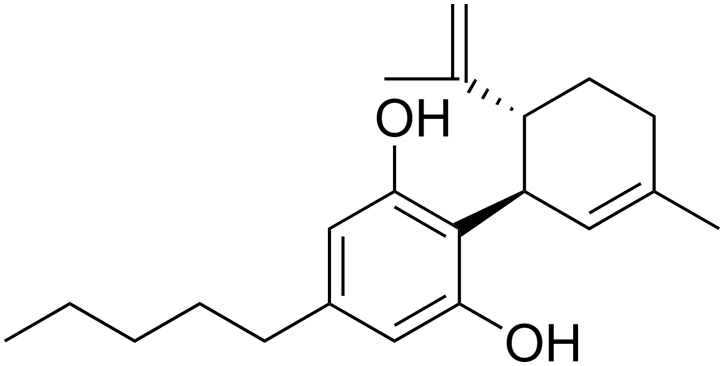
Structure of cannabidiol.

Recently, it was reported that cannabidiol (Fig. 12) has an impact on cytokine storm associated with ARDS induced by simulated infection [149].

Cannabidiol, which comes from the cannabis species, is not a psychoactive compound, but has many desirable biological activities. It has analgesic, anti-inflammatory, anti-cancer and chemopreventive properties. Cannabidiol was shown to block IL-6, one of the pro-inflammatory cytokines, in models of inflammatory diseases [150]. Massive production of interleukins IL-6, IL-1β, and IL-17 is associated with the occurrence of cytokine storm in patients with severe COVID-19. Nasal administration of a synthetic double-stranded RNA viral analogue (Poly (I:C)) was shown to simulate the symptoms of severe infections causing ARDS and cytokine storm [149]. They also showed that administration of cannabidiol downregulates the level of pro-inflammatory cytokines and ameliorates the clinical symptoms of Poly I:C-induced ARDS. These results suggest that cannabidiol can play a protective role during ARDS, and that its using for treatment of COVID-19 can be considered. However, further studies are required to evaluate the cannabidiol therapeutic potential against SARS-CoV-2.

## 9 Summary and perspectives

Since its first detection, our knowledge about SARS-CoV-2 has been becoming increasingly comprehensive. This virus and the disease it causes, COVID-19, have dominated the work of the entire scientific world in an unprecedented global effort. Despite this, there is still no drug specifically aimed at COVID-19 treatment. Such a situation should not be surprising; development of new drugs is not a simple process that requires several safety efficacy analyses which are time-consuming. What is more, SARS-CoV-2 inoculation into the host cells makes this process even more challenging due to the fact that potential drugs cannot cause strong site effects to the host. On the other hand, there is the urgent need for developing remedial agents that could be applied to overcome the pandemic. For solving these problems, knowledge about SARS-CoV-2 pathobiology is necessary. Fortunately, researchers have already gained some insights. Due to their huge efforts, molecular details of infection mechanism, ways of the virus transmission between people, as well as in which it affects the human body are currently known. However, SARS-CoV-2 infects not only a respiratory system but also blood vessels, causing thrombosis [151] and stroke [152]. COVID-19 can result in gastrointestinal, neurological, renal, and cardiovascular complications. Numerous druggable targets associated with the virus' main structural proteins and enzymes implicated in the key steps of its life cycle were indicated, and various drugs are used for COVID-19 treatment. Yet, the effectiveness of these pharmaceuticals is far from being satisfactory. Among the different therapeutic approaches, we want to stress the strategy that indicates an increase in the involvement of deaminases belonging to ADAR and particularly to APOBEC group, in editing of the SARS-CoV-2 RNA. Such a strategy could further reduce the already low cytosine content in the viral genome, leading to the loss of its integrity and, in a consequence to defeating the virus. Also, strategies based on nucleic acid technologies can be proven to be useful due to the ability of therapeutic nucleotides to inhibit translation by targeting genes. These approaches have huge potential; however, the designing and preparation of appropriate siRNA, ribozymes or ASO is a complicated process due to the need to ensure the stability of the synthesized nucleic acids and to develop modes of their delivery to the selected target. With regard to specific drugs against SARS-CoV-2, natural products could be a good base for their development. Bioactive natural compounds present in medical plants can be used as supportive substances in COVID-19 treatment but their structures may also serve as scaffolds for designing new therapeutic agents. Curcumin, a natural compound with high safety and low toxicity, which shows a lot of desired biological activities, can be considered a good candidate.

Lately, it has been shown that the severe, life-threatening course of SARS-CoV-2 pneumonia may be due to an inborn defect in the immune system in which the production of type I interferon is impaired [153]. This suggests that the administration of interferon may provide some therapeutic benefits to patients with immune defect. It was also found that some of the patients with severe COVID-19 have neutralizing auto-antibodies against type I interferon [154]. Similarly to inborn errors of type I interferon production, presence of the neutralizing auto-antibodies results in devastating diseases. In this case, therapy with interferon β may help, as the presence of autoantibodies to this interferon is very rare in patients with antibodies to type I interferon [154].

Waiting for progress in COVID-19 treatment, we should be aware that our behavior, i.e., wearing of mask, hand washing and social distancing can make a significant impact on the reduction of the disease transmission.

## Declaration of competing interest

The authors declare that there are no conflicts of interest.

## References

[1] Hudson C.B., Beaudette F.R. (1932). Infection of the cloaca with the virus of Infectious bronchitis. Science.

[2] Hamre D., Procknow J.J. (1966). A new virus isolated from the human respiratory tract. Proc. Soc. Exp. Biol. Med..

[3] McIntosh K., Dees J.H., Becker W.B. (1967). Recovery in tracheal organ cultures of novel viruses from patients with respiratory disease. Proc. Natl. Acad. Sci. U. S. A..

[4] Wang C., Horby P.W., Hayden F.G. (2020). A novel coronavirus outbreak of global health concern. Lancet.

[5] Chang C., Lo S.-C., Wang Y.-S. (2016). Recent insights into the development of therapeutics against coronavirus diseases by targeting N protein. Drug Discov. Today.

[6] Matoba Y., Abiko C., Ikeda T. (2015). Detection of the human coronavirus 229E, HKU1, NL63, and OC43 between 2010 and 2013 in Yamagata, Japan. Jpn. J. Infect. Dis..

[7] Gaunt E.R., Hardie A., Claas E.C.J. (2010). Epidemiology and clinical presentations of the four human coronaviruses 229E, HKU1, NL63, and OC43 detected over 3 years using a novel multiplex real-time PCR method. J. Clin. Microbiol..

[8] Kuiken T., Fouchier R.A.M., Schutten M. (2003). Newly discovered coronavirus as the primary cause of severe acute respiratory syndrome. Lancet.

[9] Hilgenfeld R. (2014). From SARS to MERS: crystallographic studies on coronaviral proteases enable antiviral drug design. FEBS J..

[10] Hilgenfeld R., Peiris M. (2013). From SARS to MERS: 10 years of research on highly pathogenic human coronaviruses. Antivir. Res..

[11] Zaki A.M., van Boheemen S., Bestebroer T.M. (2012). Isolation of a novel coronavirus from a man with pneumonia in Saudi Arabia. N. Engl. J. Med..

[12] Song Z., Xu Y., Bao L. (2019). From SARS to MERS, thrusting coronaviruses into the spotlight. Viruses.

[13] Guy R.K., DiPaola R.S., Romanelli F. (2020). Rapid repurposing of drugs for COVID-19. Science.

[14] Tang B., Bragazzi N.L., Li Q. (2020). An updated estimation of the risk of transmission of the novel coronavirus (2019-nCov). Infect. Dis. Model..

[15] Zhu N., Zhang D., Wang W. (2020). China novel coronavirus investigating and research team, a novel coronavirus from patients with pneumonia in China, 2019. N. Engl. J. Med..

[16] Rossi J.J., Rossi D. (2020). Oligonucleotides and the COVID-19 pandemic: a perspective. Nucleic Acid Therapeut..

[17] J Alsaadi E.A., Jones I.M. (2019). Membrane binding proteins of coronaviruses. Future Virol..

[18] Masters P.S. (2006).

[19] de Wilde A.H., Snijder E.J., Kikkert M. (2018).

[20] Liu C., Zhou Q., Li Y. (2020). Research and development on therapeutic agents and vaccines for COVID-19 and related human coronavirus diseases. ACS Cent. Sci..

[21] Payne S. (2017). Family Coronaviridae. Viruses.

[22] Jiang S., Hillyer C., Du L. (2020). Neutralizing antibodies against SARS-CoV-2 and other human coronaviruses. Trends Immunol..

[23] Rabi F.A., Al Zoubi M.S., Kasasbeh G.A. (2020). SARS-CoV-2 and coronavirus disease 2019: what we know so far. Pathogens.

[24] Walls A.C., Park Y.-J., Tortorici M.A. (2020). Structure, function, and antigenicity of the SARS-CoV-2 spike glycoprotein. Cell.

[25] Panda P.K., Arul M.N., Patel P. (2020). Structure-based drug designing and immunoinformatics approach for SARS-CoV-2. Sci. Adv..

[26] Hikmet F., Méar L., Edvinsson Å. (2020). The protein expression profile of ACE2 in human tissues. Mol. Syst. Biol..

[27] Cantuti-Castelvetri L., Ojha R., Pedro L.D. (2020). Neuropilin-1 facilitates SARS-CoV-2 cell entry and infectivity. Science.

[28] Guo G., Ye L., Pan K. (2020). New insights of emerging SARS-CoV-2: epidemiology, etiology, clinical features, clinical treatment, and prevention. Front. Cell Dev. Biol..

[29] Shereen M.A., Khan S., Kazmi A. (2020). COVID-19 infection: origin, transmission, and characteristics of human coronaviruses. J. Adv. Res..

[30] Astuti I. (2020). Ysrafil, severe acute respiratory syndrome coronavirus 2 (SARS-CoV-2): an overview of viral structure and host response. Diabetes Metab. Syndr..

[31] Wu F., Zhao S., Yu B. (2020). A new coronavirus associated with human respiratory disease in China. Nature.

[32] Ludwig S., Zarbock A. (2020). Coronaviruses and SARS-CoV-2: a brief overview. Anesth. Analg..

[33] Dagur H.S., Dhakar S. (2020). Genome organization of covid-19 and emerging severe acute respiratory syndrome covid-19 outbreak: a pandemic. Eurasian J. Med. Oncol..

[34] Naqvi A.A.T., Fatima K., Mohammad T. (2020). Insights into SARS-CoV-2 genome, structure, evolution, pathogenesis and therapies: structural genomics approach. Biochim. Biophys. Acta (BBA) - Mol. Basis Dis..

[35] Ceraolo C., Giorgi F.M. (2020). Genomic variance of the 2019-nCoV coronavirus. J. Med. Virol..

[36] Wu A., Peng Y., Huang B. (2020). Genome composition and divergence of the novel coronavirus (2019-nCoV) originating in China. Cell Host Microbe.

[37] Bojkova D., Klann K., Koch B. (2020). Proteomics of SARS-CoV-2-infected host cells reveals therapy targets. Nature.

[38] Davidson A.D., Williamson M.K., Lewis S. (2020). Characterisation of the transcriptome and proteome of SARS-CoV-2 reveals a cell passage induced in-frame deletion of the furin-like cleavage site from the spike glycoprotein. Genome Med..

[39] Finkel Y., Mizrahi O., Nachshon A. (2021). The coding capacity of SARS-CoV-2. Nature.

[40] Dai W., Zhang B., Jiang X.-M. (2020). Structure-based design of antiviral drug candidates targeting the SARS-CoV-2 main protease. Science.

[41] Zhang L., Lin D., Sun X. (2020). Crystal structure of SARS-CoV-2 main protease provides a basis for design of improved α-ketoamide inhibitors. Science.

[42] Ullrich S., Nitsche C. (2020). The SARS-CoV-2 main protease as drug target. Bioorg. Med. Chem. Lett.

[43] Estrada E. (2020). Topological analysis of SARS CoV-2 main protease. Chaos Interdiscip. J. Nonlinear Sci..

[44] Muramatsu T., Kim Y.-T., Nishii W. (2013). Autoprocessing mechanism of severe acute respiratory syndrome coronavirus 3C-like protease (SARS-CoV 3CLpro) from its polyproteins. FEBS J..

[45] Pillaiyar T., Manickam M., Namasivayam V. (2016). An overview of Severe Acute Respiratory Syndrome–Coronavirus (SARS-CoV) 3CL protease inhibitors: peptidomimetics and small molecule chemotherapy. J. Med. Chem..

[46] Tahir ul Qamar M., Alqahtani S.M., Alamri M.A. (2020). Structural basis of SARS-CoV-2 3CLpro and anti-COVID-19 drug discovery from medicinal plants. J. Pharm. Anal..

[47] Jin Z., Du X., Xu Y. (2020). Structure of M pro from SARS-CoV-2 and discovery of its inhibitors. Nature.

[48] Cai Y., Zhang J., Xiao T. (2020). Distinct conformational states of SARS-CoV-2 spike protein. Science.

[49] Wrapp D., Wang N., Corbett K.S. (2020). Cryo-EM structure of the 2019-nCoV spike in the prefusion conformation. Science.

[50] Wang Q., Qiu Y., Li J.-Y. (2020). A unique protease cleavage sPredicted in the spike protein of the novel pneumonia coronavirus (2019-nCoV) potentially related to viral transmissibility. Virol. Sin..

[51] Hoffmann M., Kleine-Weber H., Pöhlmann S. (2020). A multibasic cleavage site in the spike protein of SARS-CoV-2 is essential for infection of human lung cells. Mol. Cell..

[52] Shang J., Wan Y., Luo C. (2020). Cell entry mechanisms of SARS-CoV-2. Proc. Natl. Acad. Sci. U.S.A..

[53] Hoffmann M., Kleine-Weber H., Schroeder S. (2020). SARS-CoV-2 cell entry depends on ACE2 and TMPRSS2 and is blocked by a clinically proven protease inhibitor. Cell.

[54] Toelzer C., Gupta K., Yadav S.K.N. (2020). Free fatty acid binding pocket in the locked structure of SARS-CoV-2 spike protein. Science.

[55] Plante J.A., Liu Y., Liu J. (2021). Spike mutation D614G alters SARS-CoV-2 fitness. Nature.

[56] Yin C. (2020). Genotyping coronavirus SARS-CoV-2: methods and implications. Genomics.

[57] Yin W., Mao C., Luan X. (2020). Structural basis for inhibition of the RNA-dependent RNA polymerase from SARS-CoV-2 by remdesivir. Science.

[58] Ahmad J., Ikram S., Ahmad F. (2020). SARS-CoV-2 RNA dependent RNA polymerase (RdRp) – a drug repurposing study. Heliyon.

[59] Gao Y., Yan L., Huang Y. (2020). Structure of the RNA-dependent RNA polymerase from COVID-19 virus. Science.

[60] Hillen H.S., Kokic G., Farnung L. (2020). Structure of replicating SARS-CoV-2 polymerase. Nature.

[61] Kirchdoerfer R.N., Ward A.B. (2019). Structure of the SARS-CoV nsp 12 polymerase bound to nsp 7 and nsp 8 co-factors. Nat. Commun..

[62] Shin D., Mukherjee R., Grewe D. (2020). Papain-like protease regulates SARS-CoV-2 viral spread and innate immunity. Nature.

[63] Devaraj S.G., Wang N., Chen Z. (2007). Regulation of IRF-3-dependent innate immunity by the papain-like protease domain of the severe acute respiratory syndrome coronavirus. J. Biol. Chem..

[64] Báez-Santos Y.M., John S.E. St, Mesecar A.D. (2015). The SARS-coronavirus papain-like protease: structure, function and inhibition by designed antiviral compounds. Antivir. Res..

[65] Liang Y., Wang M.-L., Chien C.-S. (2020). Highlight of immune pathogenic response and hematopathologic effect in SARS-CoV, MERS-CoV, and SARS-Cov-2 infection. Front. Immunol..

[66] Schoeman D., Fielding B.C. (2019). Coronavirus envelope protein: current knowledge. Virol. J..

[67] Neuman B.W., Kiss G., Kunding A.H. (2011). A structural analysis of M protein in coronavirus assembly and morphology. J. Struct. Biol..

[68] Ahmed S.F., Quadeer A.A., McKay M.R. (2020). Preliminary identification of potential vaccine targets for the COVID-19 coronavirus (SARS-CoV-2) based on SARS-CoV immunological studies. Viruses.

[69] McBride R., van Zyl M., Fielding B.C. (2014). The coronavirus nucleocapsid is a multifunctional protein. Viruses.

[70] Kang S., Yang M., Hong Z. (2020). Crystal structure of SARS-CoV-2 nucleocapsid protein RNA binding domain reveals potential unique drug targeting sites. Acta Pharm. Sin. B..

[71] Shu T., Huang M., Wu D. (2020). SARS-Coronavirus-2 Nsp13 possesses NTPase and RNA helicase activities that can be inhibited by bismuth salts. Virol. Sin..

[72] Mirza M.U., Froeyen M. (2020). Structural elucidation of SARS-CoV-2 vital proteins: computational methods reveal potential drug candidates against main protease, Nsp12 polymerase and Nsp13 helicase. J. Pharm. Anal..

[73] Ren Y., Shu T., Wu D. (2020). The ORF3a protein of SARS-CoV-2 induces apoptosis in cells. Cell. Mol. Immunol..

[74] Li J.-Y., Liao C.-H., Wang Q. (2020). The ORF6, ORF8 and nucleocapsid proteins of SARS-CoV-2 inhibit type I interferon signaling pathway. Virus Res..

[75] Flower T.G., Buffalo C.Z., Hooy R.M. (2021). Structure of SARS-CoV-2 ORF8, a rapidly evolving immune evasion protein. Proc. Natl. Acad. Sci. U. S. A..

[76] Miao G., Zhao H., Li Y. (2021). ORF3a of the COVID-19 virus SARS-CoV-2 blocks HOPS complex-mediated assembly of the SNARE complex required for autolysosome formation. Dev. Cell.

[77] Narayanan K., Huang C., Makino S. (2008). SARS coronavirus accessory proteins. Virus Res..

[78] McBride R., Fielding B.C. (2012). The role of severe acute respiratory syndrome (SARS)-Coronavirus accessory proteins in virus pathogenesis. Viruses.

[79] Zinzula L. (2021). Lost in deletion: the enigmatic ORF8 protein of SARS-CoV-2. Biochem. Biophys. Res. Commun..

[80] Arya R., Kumari S., Pandey B. (2021). Structural insights into SARS-CoV-2 proteins. J. Mol. Biol..

[81] Majumdar P., Niyogi S. (2020). ORF3a mutation associated with higher mortality rate in SARS-CoV-2 infection. Epidemiol. Infect..

[82] Hachim A., Kavian N., Cohen C.A. (2020). ORF8 and ORF3b antibodies are accurate serological markers of early and late SARS-CoV-2 infection. Nat. Immunol..

[83] Lei X., Dong X., Ma R. (2020). Activation and evasion of type I interferon responses by SARS-CoV-2. Nat. Commun..

[84] Pereira F. (2020). Evolutionary dynamics of the SARS-CoV-2 ORF8 accessory gene. Infect. Genet. Evol..

[85] Addetia A., Xie H., Roychoudhury P. (2020). Identification of multiple large deletions in ORF7a resulting in in-frame gene fusions in clinical SARS-CoV-2 isolates. J. Clin. Virol..

[86] Pancer K., Milewska A., Owczarek K. (2020). The SARS-CoV-2 ORF10 is not essential in vitro or in vivo in humans. PLoS Pathog..

[87] Wang I.X., So E., Devlin J.L. (2013). ADAR regulates RNA editing, transcript stability, and gene expression. Cell Rep..

[88] Rueter S.M., Burns C.M., Coode S.A. (1995). Glutamate receptor RNA editing in vitro by enzymatic conversion of adenosine to inosine. Science.

[89] Yang J.H., Sklar P., Axel R. (1995). Editing of glutamate receptor subunit B pre-mRNA in vitro by site-specific deamination of adenosine. Nature.

[90] Nabel C.S., Manning S.A., Kohli R.M. (2012). The curious chemical biology of cytosine: deamination, methylation, and oxidation as modulators of genomic potential. ACS Chem. Biol..

[91] Sah R., Rodriguez-Morales A.J., Jha R. (2020). Complete genome sequence of a 2019 novel coronavirus (SARS-CoV-2) strain Isolated in Nepal. Microbiol. Resour. Announc..

[92] Pyrc K., Jebbink M.F., Berkhout B. (2004). Genome structure and transcriptional regulation of human coronavirus NL63. Virol. J..

[93] Giorgio S.D., Martignano F., Torcia M.G. (2020). Evidence for host-dependent RNA editing in the transcriptome of SARS-CoV-2. Sci. Adv..

[94] Sinha N., Balayla G. (2020). Hydroxychloroquine and covid-19. Postgrad. Med..

[95] Choudhary R., Sharma A.K. (2020). Potential use of hydroxychloroquine, ivermectin and azithromycin drugs in fighting COVID-19: trends, scope and relevance. New Microbes New Infect.

[96] Andreani J., Le Bideau M., Duflot I. (2020). In vitro testing of combined hydroxychloroquine and azithromycin on SARS-CoV-2 shows synergistic effect. Microb. Pathog..

[97] Siddiqui A.J., Jahan S., Ashraf S.A. (2020). Current status and strategic possibilities on potential use of combinational drug therapy against COVID-19 caused by SARS-CoV-2. J. Biomol. Struct. Dyn..

[98] Liu J., Cao R., Xu M. (2020). Hydroxychloroquine, a less toxic derivative of chloroquine, is effective in inhibiting SARS-CoV-2 infection in vitro. Cell Discov.

[99] Ferner R.E., Aronson J.K. (2020). Chloroquine and hydroxychloroquine in covid-19. BMJ.

[100] Geleris J., Sun Y., Platt J. (2020). Observational study of hydroxychloroquine in hospitalized patients with Covid-19. N. Engl. J. Med..

[101] Asai A., Konno M., Ozaki M. (2020). COVID-19 drug discovery using intensive approaches. Int. J. Mol. Sci..

[102] Huang D., Yu H., Wang T. (2021). Efficacy and safety of umifenovir for coronavirus disease 2019 (COVID-19): a systematic review and meta-analysis. J. Med. Virol..

[103] Wu R., Wang L., Kuo H.-C.D. (2020). An update on current therapeutic drugs treating COVID-19. Curr. Pharmacol. Rep..

[104] Villalaín J. (2010). Membranotropic effects of arbidol, a broad anti-viral molecule, on phospholipid model membranes. J. Phys. Chem. B.

[105] Leneva I.A., Russell R.J., Boriskin Y.S. (2009). Characteristics of arbidol-resistant mutants of influenza virus: implications for the mechanism of anti-influenza action of arbidol. Antivir. Res..

[106] Wang X., Cao R., Zhang H. (2020). The anti-influenza virus drug, arbidol is an efficient inhibitor of SARS-CoV-2 in vitro. Cell Discov.

[107] Lian N., Xie H., Lin S. (2020). Umifenovir treatment is not associated with improved outcomes in patients with coronavirus disease 2019: a retrospective study. Clin. Microbiol. Infect..

[108] Aktaş A., Tüzün B., Aslan R. (2020). New anti-viral drugs for the treatment of COVID-19 instead of favipiravir. J. Biomol. Struct. Dyn..

[109] Lu C.-C., Chen M.-Y., Lee W.-S. (2020). Potential therapeutic agents against COVID-19: what we know so far. J. Chin. Med. Assoc..

[110] Hashemian S.M., Farhadi T., Velayati A.A. (2020). A review on remdesivir: a possible promising agent for the treatment of COVID-19. Drug Des. Dev. Ther..

[111] Siegel D., Hui H.C., Doerffler E. (2017). Discovery and synthesis of a phosphoramidate prodrug of a pyrrolo[2,1-f][triazin-4-amino] adenine C-nucleoside (GS-5734) for the treatment of ebola and emerging viruses. J. Med. Chem..

[112] Gordon C.J., Tchesnokov E.P., Woolner E. (2020). Remdesivir is a direct-acting antiviral that inhibits RNA-dependent RNA polymerase from severe acute respiratory syndrome coronavirus 2 with high potency. J. Biol. Chem..

[113] Tchesnokov E.P., Feng J.Y., Porter D.P. (2019). Mechanism of inhibition of Ebola virus RNA-dependent RNA polymerase by remdesivir. Viruses.

[114] Agostini M.L., Andres E.L., Sims A.C. (2018). Coronavirus susceptibility to the antiviral remdesivir (GS-5734) is mediated by the viral polymerase and the proofreading exoribonuclease. mBio.

[115] Brown A.J., Won J.J., Graham R.L. (2019). Broad spectrum antiviral remdesivir inhibits human endemic and zoonotic deltacoronaviruses with a highly divergent RNA dependent RNA polymerase. Antivir. Res..

[116] Sheahan T.P., Sims A.C., Graham R.L. (2017). Broad-spectrum antiviral GS-5734 inhibits both epidemic and zoonotic coronaviruses. Sci. Transl. Med..

[117] Sheahan T.P., Sims A.C., Leist S.R. (2020). Comparative therapeutic efficacy of remdesivir and combination lopinavir, ritonavir, and interferon beta against MERS-CoV. Nat. Commun..

[118] Beigel J.H., Tomashek K.M., Dodd L.E. (2020). Remdesivir for the treatment of Covid-19 — preliminary report. N. Engl. J. Med..

[119] (2020). Dexamethasone in hospitalized patients with covid-19 — preliminary report. N. Engl. J. Med..

[120] Duan K., Liu B., Li C. (2020). Effectiveness of convalescent plasma therapy in severe COVID-19 patients. Proc. Natl. Acad. Sci. U.S.A..

[121] Shih H.-I., Wu C.-J., Tu Y.-F. (2020). Fighting COVID-19: a quick review of diagnoses, therapies, and vaccines. Biomed. J..

[122] Marovich M., Mascola J.R., Cohen M.S. (2020). Monoclonal antibodies for prevention and treatment of COVID-19. J. Am. Med. Assoc..

[123] Asha K., Kumar P., Sanicas M. (2019). Advancements in nucleic acid based therapeutics against respiratory viral infections. J. Clin. Med..

[124] Agrawal N., Dasaradhi P.V.N., Mohmmed A. (2003). RNA interference: biology, mechanism, and applications. Microbiol. Mol. Biol. Rev..

[125] Wang Z., Ren L., Zhao X. (2004). Inhibition of severe acute respiratory syndrome virus replication by small interfering RNAs in mammalian cells. J. Virol..

[126] Wu C.-J., Huang H.-W., Liu C.-Y. (2005). Inhibition of SARS-CoV replication by siRNA. Antivir. Res..

[127] Zhang Y., Li T., Fu L. (2004). Silencing SARS-CoV spike protein expression in cultured cells by RNA interference. FEBS Lett..

[128] Fukushima A., Fukuda N., Lai Y. (2009). Development of a chimeric DNA-RNA hammerhead ribozyme targeting SARS virus. Intervirology.

[129] Bennett C.F., Swayze E.E. (2010). RNA targeting therapeutics: molecular mechanisms of antisense oligonucleotides as a therapeutic platform. Annu. Rev. Pharmacol. Toxicol..

[130] Neuman B.W., Stein D.A., Kroeker A.D., Perlman S., Holmes K.V. (2006). The Nidoviruses.

[131] Neuman B.W., Stein D.A., Kroeker A.D. (2005). Inhibition, escape, and attenuated growth of Severe Acute Respiratory Syndrome Coronavirus treated with antisense morpholino oligomers. J. Virol..

[132] Kruse R.L. (2020). Therapeutic strategies in an outbreak scenario to treat the novel coronavirus originating in Wuhan, China. F1000Research..

[133] Sayed A.M., Alhadrami H.A., El-Gendy A.O. (2020). Microbial natural products as potential inhibitors of SARS-CoV-2 main protease (Mpro). Microorganisms.

[134] Antonio A. da S., Wiedemann L.S.M., Veiga-Junior V.F. (2020). Natural products role against COVID-19. RSC Adv..

[135] Wen C.-C., Kuo Y.-H., Jan J.-T. (2007). Specific plant terpenoids and lignoids possess potent antiviral activities against Severe Acute Respiratory Syndrome Coronavirus. J. Med. Chem..

[136] Joshi R.S., Jagdale S.S., Bansode S.B. (2021). Discovery of potential multi-target-directed ligands by targeting host-specific SARS-CoV-2 structurally conserved main protease. J. Biomol. Struct. Dyn..

[137] Liu Z., Ying Y. (2020). The inhibitory effect of curcumin on virus-induced cytokine storm and its potential use in the associated severe pneumonia. Front. Cell Dev. Biol..

[138] Dai J., Gu L., Su Y. (2018). Inhibition of curcumin on influenza A virus infection and influenzal pneumonia via oxidative stress, TLR2/4, p38/JNK MAPK and NF-κB pathways. Int. Immunopharm..

[139] Zhang B., Swamy S., Balijepalli S. (2019). Direct pulmonary delivery of solubilized curcumin reduces severity of lethal pneumonia. FASEB J.

[140] Chen L., Lu Y., Zhao L. (2018). Curcumin attenuates sepsis-induced acute organ dysfunction by preventing inflammation and enhancing the suppressive function of Tregs. Int. Immunopharm..

[141] Chai Y., Chen Y., Lin S. (2020). Curcumin regulates the differentiation of naïve CD4+T cells and activates IL-10 immune modulation against acute lung injury in mice. Biomed. Pharmacother..

[142] Ren Y., Yang Z., Sun Z. (2019). Curcumin relieves paraquat-induced lung injury through inhibiting the thioredoxin interacting protein/NLR pyrin domain containing 3-mediated inflammatory pathway. Mol. Med. Rep..

[143] Pannu N., Bhatnagar A. (2019). Resveratrol: from enhanced biosynthesis and bioavailability to multitargeting chronic diseases. Biomed. Pharmacother..

[144] Zupančič Š., Lavrič Z., Kristl J. (2015). Stability and solubility of trans-resveratrol are strongly influenced by pH and temperature. Eur. J. Pharm. Biopharm..

[145] Chimento A., De Amicis F., Sirianni R. (2019). Progress to improve oral bioavailability and beneficial effects of resveratrol. Int. J. Mol. Sci..

[146] Filardo S., Di Pietro M., Mastromarino P. (2020). Therapeutic potential of resveratrol against emerging respiratory viral infections. Pharmacol. Ther..

[147] Li Y.-Q., Li Z.-L., Zhao W.-J. (2006). Synthesis of stilbene derivatives with inhibition of SARS coronavirus replication. Eur. J. Med. Chem..

[148] Wahedi H.M., Ahmad S., Abbasi S.W. (2021). Stilbene-based natural compounds as promising drug candidates against COVID-19. J. Biomol. Struct. Dyn..

[149] Khodadadi H., Salles É.L., Jarrahi A. (2020). Cannabidiol modulates cytokine storm in acute respiratory distress syndrome induced by simulated viral infection using synthetic RNA. Cannabis Cannabinoid Res..

[150] Bozkurt T.E. (2019). Endocannabinoid system in the airways. Molecules.

[151] Ackermann M., Verleden S.E., Kuehnel M. (2020). Pulmonary vascular endothelialitis, thrombosis, and angiogenesis in Covid-19. N. Engl. J. Med..

[152] Oxley T.J., Mocco J., Majidi S. (2020). Large-vessel stroke as a presenting feature of Covid-19 in the young. N. Engl. J. Med..

[153] Zhang Q., Bastard P., Liu Z. (2020). Inborn errors of type I IFN immunity in patients with life-threatening COVID-19. Science.

[154] Bastard P., Rosen L.B., Zhang Q. (2020). Auto-antibodies against type I IFNs in patients with life-threatening COVID-19. Science.

